# General risk preference comes up short when predicting risk-taking frequency

**DOI:** 10.1038/s41598-026-36713-w

**Published:** 2026-01-23

**Authors:** Maja Asp, Marielle Abed, Philip Millroth

**Affiliations:** Department of Psychology, Uppsala University, P.O. Box 1225, Uppsala, SE-751 42 Sweden

**Keywords:** Risk taking, Preferences, Impulsivity, Sensation seeking, Gender, Psychology, Human behaviour

## Abstract

Situations involving risk are common in human affairs, from financial investments, criminal behavior, and health-related decisions. Peoples’ self-reported risk preferences, where individuals in various ways report how they feel about risk, have been considered central to understanding why, and predict when, people behave differently in real-life situations involving risk. However, various other factors have also been suggested as predictors, including age, gender, education, income, anxiety, sensation seeking, impulsivity, and personality traits like neuroticism and extraversion. Still, research is limited on which factors best predict the frequency of risk-taking in real life. In this study, we asked respondents (*n* = 760) to report how often they engaged in various risk-taking behaviors along with the abovementioned variables with the aim of predicting risk-taking frequency. The results from Bayesian multi-model inference analyses showed that the most important predictors of risk-taking frequency were impulsivity, sensation seeking, health and social risk preferences, and gender. The study highlights the importance of examining multiple variables simultaneously when predicting risk-taking frequency. The importance of the concept of general risk-preference, which occupies a central role in many influential theories of risk-taking, should arguably be reconsidered.

**Supplementary Information:**

The online version contains supplementary material available at 10.1038/s41598-026-36713-w.

## Introduction

Risk – referring to situations with known outcome variance or behaviors with short-term rewards but potential downsides^1–5^- is an inherent part of human life, shaping decisions that range from the mundane to the extraordinary. Whether deciding to invest in the stock market, partake in extreme sports like bungee jumping, or engage in behaviors like smoking, individuals constantly navigate uncertain outcomes. Mapping and understanding human behavior in situations involving risk has long been a central research question in the social sciences resulting in several prominent theories, both normative (expected utility theory^6^: and descriptive (e.g., [cumulative]prospect theory^7,8^:, and for understanding clinical outcomes (*4*,*5*). One variable that contemporary researchers have suggested is critical for predicting an individual’s engagement in risk-taking behavior is risk preference—that is, whether a person is attracted to risks or not^9–11^. From this perspective, individuals who are more attracted to risks should also engage more in risk-taking behaviors^12,13^.

However, risk preferences measured by self-assessment of how people feel about risk, stated risk-preferences (SRPs), have shown mixed results regarding to how it is associated with the extent that people actually engage in risk-taking behavior^12,14^. In addition, there are discussions about whether it is best to use general or domain-specific risk preference in research^15,16^. For example, it has been argued that both general and domain-specific risk preference can predict risk-taking frequency and that the type of behavior to be investigated should govern which of them should be used^15^.

Even more critically, the predictive ability of SRPs have not been directly compared to that of relevant associated constructs. In addition to risk preference, other factors have indeed been shown to play a role in the number of risk-taking behaviors people engage in outside of the lab, including age, gender, education, income, anxiety, sensation seeking, impulsivity, and personality traits (extraversion, neuroticism). We do not claim that those variables constitute an exhaustive list of such factors – however, in our opinion, they are the most prominent ones suggested in the literature that explicitly focus on understanding risk-taking behaviors outside of the lab.

Age plays a critical role in frequency of risk-taking behavior and follows an inverted U-shaped curve, being low in childhood, increases during puberty, peaks in adolescence and early adulthood, and declines in later adulthood^17,18^. This trend can be explained by the development of the prefrontal cortex (PFC), which matures gradually into early adulthood and is responsible for cognitive control, decision-making, and inhibition^19^. Adolescents and young adults are more likely to engage in risk-taking behaviors such as high-speed driving, smoking, and binge drinking because their cognitive control systems are still developing, while their emotional and affective systems are more sensitive to rewards and excitement^20^. As individuals age, improved cognitive control reduces their propensity to take risks.

Gender differences in risk-taking are well-documented, with men engaging in higher levels of risk-taking frequency compared to women across domains like substance use, gambling, and financial investments^21,22^. Social norms and gender identity further amplify these differences. For example, men are often socialized to view risk-taking as a marker of masculinity, particularly behaviors such as heavy alcohol consumption and thrill-seeking^23,24^. Women, on the other hand, are more likely to take risks in social and career-related domains where they feel more confident and value success^22^. Gendered media portrayals also influence behavior, with alcohol consumption depicted as socially desirable for men but stigmatized for women^25^.

A negative relationship exists between educational level and risk-taking frequency. Individuals with lower educational attainment are more likely to engage in risky behaviors such as smoking, substance use, violence, and unprotected sex^26,27^. For instance, adolescents with lower education levels exhibit higher rates of sexual risk-taking and substance use compared to their more educated peers. One explanation is that lower education may be part of a broader cluster of problem behaviors that share common risk and protective factors, such as lack of support or impulsive tendencies^28^.

Similarly, income has a negative correlation with risk-taking across various domains. Individuals with lower income levels tend to engage more frequently in health risks like smoking and alcohol abuse, as well as financial and traffic-related risks^29–31^. A possible explanation is that individuals with fewer resources take more risks to improve their circumstances, such as taking high-interest loans to resolve debts^32^. Additionally, poverty may encourage a focus on immediate rewards, such as relaxation or social status, over long-term benefits, leading to behaviors like substance use^30^.

The relationship between anxiety and risk-taking frequency is complex, with studies reporting mixed findings. Some research suggests a negative relationship, where individuals with higher anxiety avoid risks due to cognitive biases such as heightened sensitivity to uncertainty or threat^33,34^. This manifests in risk-avoidant financial decisions, such as abstaining from investments or gambling. In contrast, other studies find a positive relationship between anxiety and risk-taking, particularly among adolescents, where risky behaviors serve as a coping mechanism to alleviate aversive emotions^35,36^. This temporary relief reinforces the behavior, contributing to increased engagement in risk-taking.

Sensation seeking is one of the strongest predictors of risk-taking frequency. Defined as the pursuit of novel, intense, and thrilling experiences, sensation seeking motivates behaviors such as extreme sports, gambling, and substance use^37^. Research consistently shows a positive relationship between sensation seeking and risk-taking across multiple domains, including health, recreational, and finances^38,39^. The excitement and reward associated with risky activities act as positive reinforcement, making sensation-seekers more likely to engage in behaviors others may avoid^40^.

Impulsivity, characterized by actions performed without forethought, also predicts higher risk-taking frequency. Impulsive individuals struggle with self-control, making them more susceptible to short-term rewards despite potential long-term negative consequences^36,41^. Behaviors such as risky driving, unprotected sex, and substance use often provide immediate pleasure or relief, reinforcing the impulsive tendencies of these individuals^40,42^. This lack of self-regulation increases their involvement in behaviors with uncertain outcomes.

Finally, personality traits like extraversion and neuroticism have been linked to risk-taking frequency. Extroverted individuals, who are social, energetic, and thrill-seeking, are more likely to take risks to pursue excitement and rewards^36^. Conversely, neurotic individuals, who experience high levels of anxiety, may engage in risk-taking as a coping strategy to reduce negative emotions temporarily^35^. However, the relationship between neuroticism and risk-taking can vary based on individuals’ ability to manage their emotions. Research suggests that combinations of personality traits are particularly important, as traits like low conscientiousness coupled with high extraversion and neuroticism predict greater risk-taking and more frequent accidents^43^.

By studying the abovementioned variables at the same time as risk preferences, their respective role in predicting the frequency of risk-taking behaviors outside laboratory settings can be clarified. Perhaps the role of risk preference needs to be neutralized and other variables emphasized as central to research? Another reason to test the predictive power of these variables is that many real-life risk-taking behaviors can have profound and often polarizing consequences, either strongly positive or strongly negative. For instance, financial investments can lead to wealth accumulation and long-term stability, but they can also result in substantial losses and financial hardship. Similarly, engaging in extreme sports may bring exhilaration, personal growth, and physical fitness, but it also carries the risk of serious injury or death. To design more effective clinical interventions that help individuals adjust their natural risk-taking tendencies—whether by encouraging beneficial risks or mitigating harmful ones—it is crucial to identify the psychological mechanisms that most strongly predict these behaviors and focus on them as targets for intervention.

Here, we address the research gap in a cross-sectional survey study (*n* = 760) in which participants reported their risk-taking frequency across several real-life behaviors, along with filling in validated self-report measures of the abovementioned correlates. Table 1 provides an overview of the included variables. More specifically, the study addresses the following research questions: (i) Which variables are the most important predictors of real-life risk-taking frequency when multiple established correlates are modeled simultaneously? (ii) Does general risk preference—which occupies a central role in many theories of risk-taking—remain an important predictor when other variables are included? (iii) Do domain-specific risk preferences predict risk-taking frequency, and if so, which domains are most important? Given the limited prior research directly comparing these predictors, we adopted an exploratory approach using Bayesian model averaging rather than testing specific directional hypotheses. (see Method).

## Results

### Descriptive statistics

**Table 1:** Provide descriptive statistics for the variables presented in the methods section. Figure 1 Provide a correlation matrix for (Kendall’s tau) for predictors used in the inference analyses. Note that this matrix is for illustrative purposes, aiding the interpreting the multi-model inferences. Thus, they Provide no hypothesis testing.

Variable	M	Md.	Min.	Max.	25th perc.	75th perc.	SD
General risk preference	17.4	16.	8.00	40.00	12.0	21.0	6.35
Domain-specific (recreational)	2.20	2.00	1.00	5.00	1.00	3.00	1.25
Domain-specific (health)	1.83	1.00	1.00	5.00	1.00	2.00	1.04
Domain-specific (career)	2.10	2.00	1.00	5.00	1.00	3.00	1.13
Domain-specific (safety)	1.86	2.00	1.00	5.00	1.00	2.00	1.03
Domain-specific (social)	2.60	2.00	1.00	5.00	2.00	4.00	1.22
Domain-specific (financial)	1.75	1.00	1.00	5.00	1.00	2.00	1.97
Anxiety	11.1	11.0	5.00	20.00	8.00	14.0	3.83
Neuroticism	5.27	5.00	2.00	10.00	4.00	7.00	1.98
Extraversion	6.88	7.00	2.00	10.00	6.00	8.00	2.07
Sensation seeking	22.3	22.0	8.00	40.00	17.0	27.0	6.70
Impulsivity	31.4	31.0	16.0	56.00	26.0	36.0	6.79
Risk-taking frequency (sum of ranks)	6789	6730	4070	10,408	5944	7574	1193

Table 1. Descriptive statistics for the non-demographic continuous variables included in the study, including means (M), medians (Md.) minimal values (Min.), maximal values (Max.) percentiles, and standard deviations (SD).

**Fig. 1:**
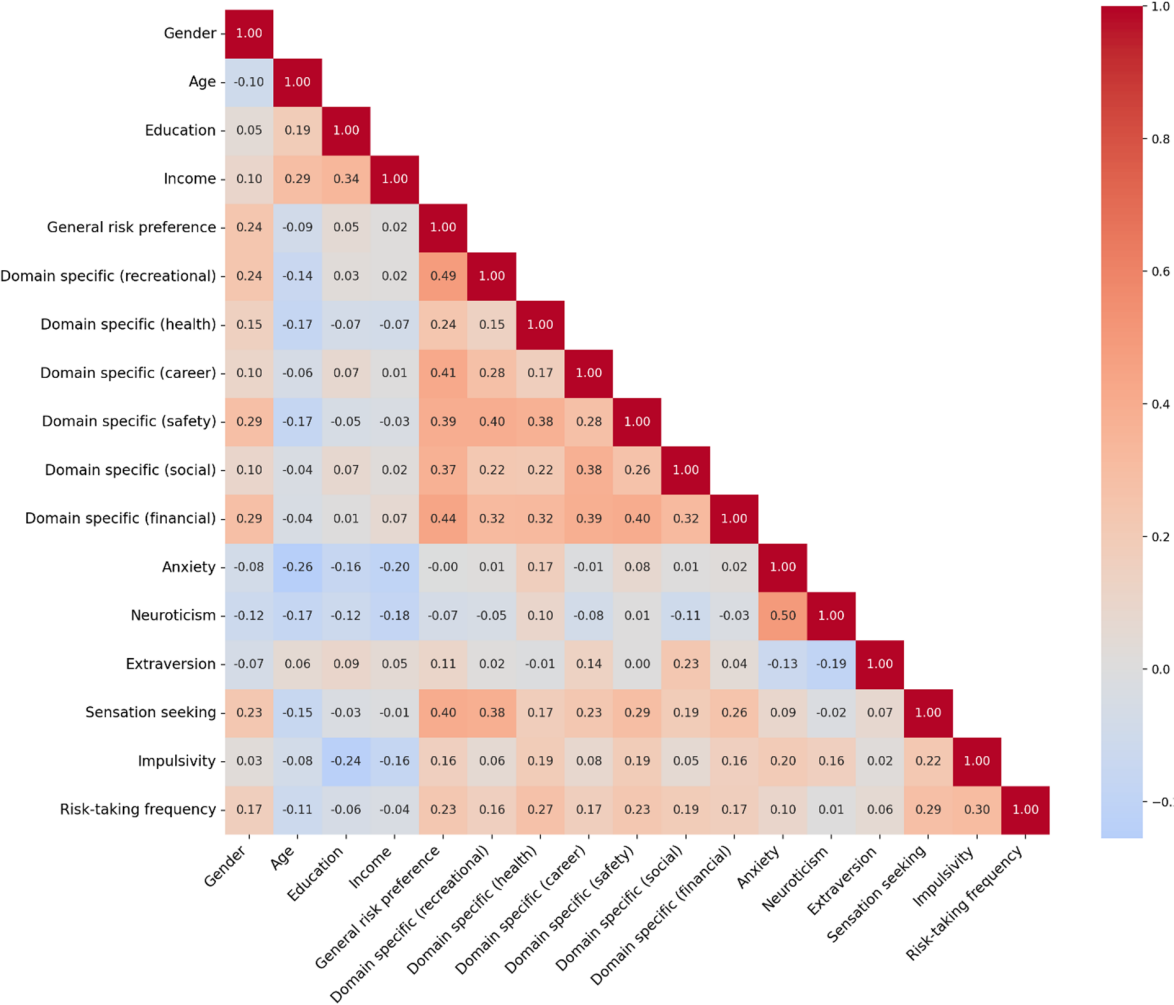
Correlation matrix of included variables. Kendall’s Tau estimates between all variables that were considered as predictors in the modeling analyses and the outcome variable (risk-taking frequency).

### Bayesian model averaging

The Bayesian regression analysis showed that impulsivity, sensation seeking, health risk preference, social risk preference, and gender are important variables for predicting risk-taking frequency. For these five variables, the posterior probabilities increased from their prior probabilities to 1.00 for impulsivity, sensation seeking, and health risk preference; 0.965 for social risk preference; and 0.918 for gender. Posterior probabilities equal to or close to 1.00 indicate that the model most likely to best predict risk-taking frequency includes these variables. The five most important variables remained consistent regardless of the prior probability settings used in JASP.

**Fig. 2:**
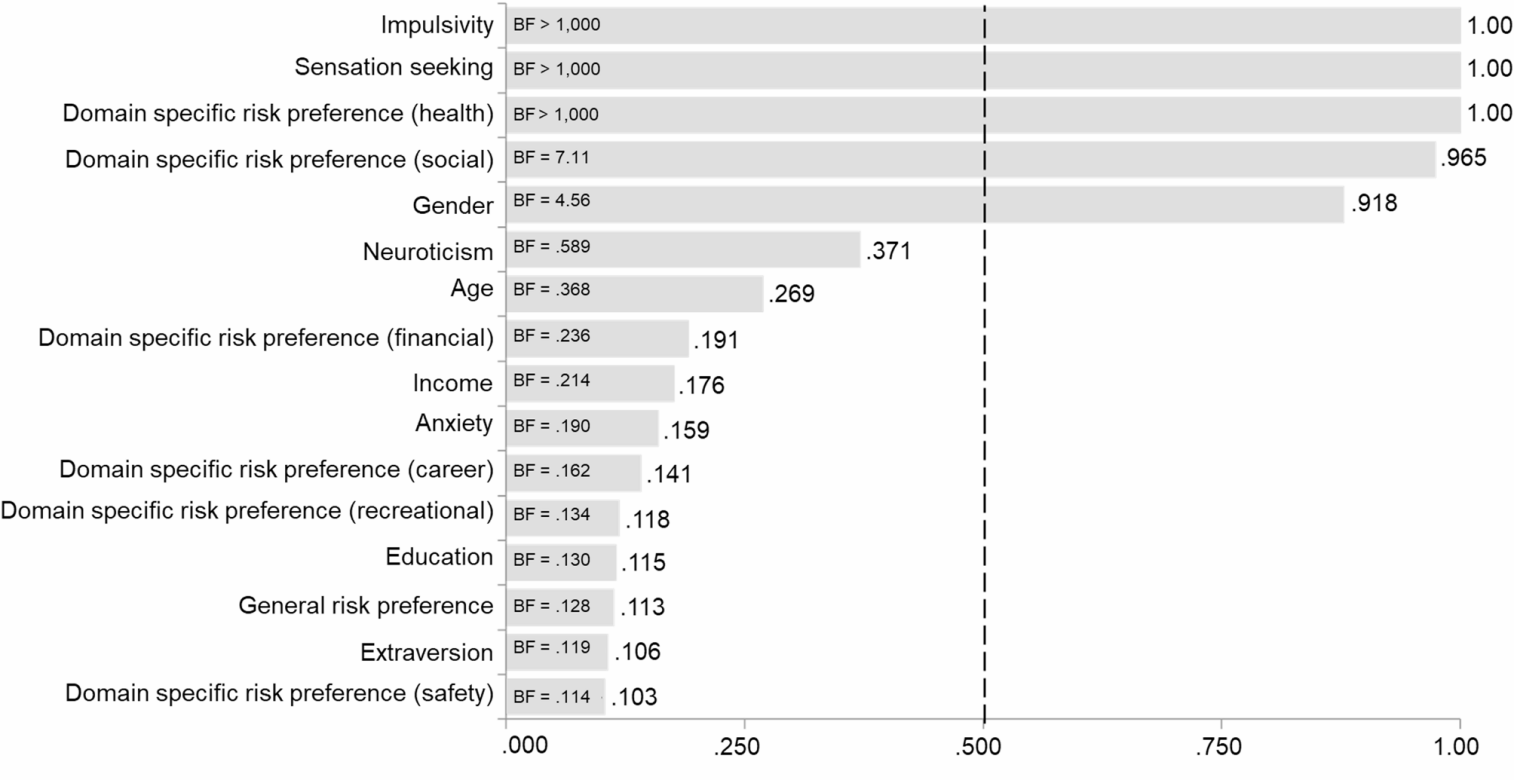
Importance of the predictors in terms of posterior inclusion probabilities. BFs depict how much more likely models that include a variable are compared to those that exclude it. Dotted line illustrates the prior inclusion probability.

The BF_inclusion_ values revealed that models including impulsivity, sensation seeking, or domain specific risk preference were > 1,000 times more likely than models without the terms, and the corresponding value was 7.11 for social risk preference and 4.56 for gender 7.23. Figure 2 illustrates the change from prior to posterior probabilities and presents the BF_inclusion_ for all variables (the full table of estimates along with averaged estimates for standardized regression coefficients are provided in Table S1 in the Supplementary Material).

Thus, the most likely variables for predicting risk-taking frequency are impulsivity, sensation seeking, health risk preference, social risk preference, and gender. Among these, impulsivity has the strongest predictive power, as its standardized regression coefficient is the highest (*β* = 0.338; *SD* = 0.033). In descending order of predictive power, the next variables are sensation seeking (*β* = 0.200; *SD* = 0.036), health risk preference (*β* = 0.185; *SD* = 0.035), social risk preference (*β* = 0.111; *SD* = 0.040), and gender (*β* = 0.087; *SD* = 0.040).

## Discussion

The purpose of this study was to investigate the potential roles of variables such as risk preference, age, gender, education level, income, anxiety, sensation seeking, impulsivity, and the personality traits extraversion and neuroticism in predicting individuals’ risk-taking frequency. The variables most likely to predict risk-taking frequency from a probabilistic perspective, when considering a comprehensive range of relevant predictive models, were impulsivity, sensation seeking, health risk preference, social risk preference, and gender. Impulsivity had the strongest predictive power, as its standardized regression coefficient was the highest. In descending order of predictive power, the next were sensation seeking, health risk preference, social risk preference, and gender.

The finding that impulsivity and sensation seeking play key roles in predicting risk-taking frequency and have the strongest predictive power aligns with previous research, which has consistently shown evidence of their relationship with risk-taking^38,44^. In this study, regression coefficients indicated a positive relationship between both impulsivity and sensation seeking and risk-taking frequency.

Theories presented in the introduction can explain why sensation-seekers and impulsive individuals tend to engage in more risk-taking behaviors than others. It has been argued that risk taking appeals to sensation-seekers because such behaviors often involve excitement^40^. Additionally, many risk-taking behaviors offer short-term positive consequences^36^, which, from a learning theory perspective, influence behavior more strongly than long-term consequences^42^. Impulsive individuals’ lack of self-control may make them more susceptible to short-term rewards, explaining their higher risk-taking frequency^36,40,42^.

Sensation seeking and impulsivity have also shown consistent evidence of correlation with risk preferences^45,46^. At the same time, prior studies have found a relationship between risk preferences and risk-taking frequency. The current study demonstrates that impulsivity and sensation seeking can predict risk-taking frequency when risk preferences are included in the analysis and that general risk preference loses its predictive power when additional variables are included. One possibility is that impulsivity and sensation seeking influence stated risk preferences, which in turn relate to behavior; alternatively, impulsivity and sensation seeking may be more proximal predictors of risk-taking, with stated general risk preference serving partly as a downstream reflection of these traits. However, the causal ordering among these variables cannot be determined from cross-sectional data. One might worry that impulsivity and risk-taking frequency overlap conceptually. However, impulsivity predicted domain-specific indices that do not obviously reflect impulsive behavior, such as health and economic risks (see Tables S4-S8), suggesting the relationship is not purely tautological.

Consistent with previous studies, the current research also shows that domain-specific risk preferences, particularly in health and social contexts, can predict risk-taking frequency^47,48^. The results also align with prior findings indicating that men engage in risk taking more frequently^22,23^. One possible explanation is that social constructions of masculinity often involve risk-taking behaviors^24,49^.

The results challenge earlier assumptions that risk preference is the central factor in predicting risk-taking frequency^9–11^. While previous research suggests that all domain-specific risk preferences have good predictive validity^47^, the current study does not support this claim. It appears more effective to focus on specific domain-specific risk preferences, as this study found that only two of the six domains (health and social) are significant in predicting risk-taking frequency, while safety, recreational, and career risk preferences showed evidence of not being important predictors. One possibility could be that health and social risks are more ubiquitous in everyday life compared to recreational risks (e.g., extreme sports) or financial risks (e.g., gambling), which require specific opportunities or resources. The everyday prevalence of health and social risk decisions may make preferences in these domains more behaviorally consequential for a general adult sample. Additionally, the results indicate that general risk preference has moderate evidence for not being a key variable, which also contradicts previous research^47,50,51^.

Beyond general risk preference and the aforementioned domains, extraversion, income, education level, and anxiety also showed moderate evidence of not being important in predicting risk-taking frequency. This contradicts prior research that found correlations between these variables and risk taking^27,30,43,52^. These findings may reflect the diminishing importance of these variables when additional predictors are included in the analysis. To test this, we conducted additional analyses not outlined in the pre-registration protocol where we assessed how likely the variables are be predictive when used as standalone predictors. Indeed, all variables except education, income, neuroticism, and extraversion should conclusive evidence when examined in isolation. The full results are presented in the Supplementary Material (Table S2).

Several limitations should be acknowledged. First, nineteen survey questions were combined into a measure of risk-taking frequency, which was deemed appropriate as previous research has suggested that these or similar behaviors represent typical risk decisions^16,53^. However, aggregating the questions into a single scale may be problematic for two reasons: (*i*) if the questions have low internal consistency, resulting in low reliability for the aggregated scale, and (*ii*) if risk-taking frequency is domain-specific rather than general, in which case it should be measured separately, as different variables might predict different domains of risk-taking.

To address concerns about psychometric properties, we conducted additional analyses not outlined in the pre-registration protocol. First, a principal component analysis (PCA) was conducted to evaluate the questions in the aggregated scale. PCA is specifically used to group items into fewer indices, unlike factor analysis, which aims to identify underlying constructs^54^. The PCA revealed that the questions from the risk-taking frequency scale grouped into five interpretable indices labeled as health, procrastination, social, safety, and economic (see Table S3 in the Supplementary Material). Each of the five identified indices included two or three questions, while five questions did not load onto any index. If the scale had unacceptably low reliability, items would not have grouped into coherent indices. Furthermore, the scale correlated in expected directions with theoretically related constructs such as impulsivity, sensation seeking, and risk preferences (see Fig. 1), providing evidence of convergent validity. Relatedly, our scale captures heterogeneous behaviors across domains and is better understood as a formative rather than reflective measure—an individual may engage heavily in one type of risk-taking (e.g., smoking) but not another (e.g., gambling), and this heterogeneity is substantively meaningful rather than indicative of poor reliability^55^. For formative measures, traditional internal consistency metrics such as Cronbach’s alpha are not appropriate and may even be misleading^46^. A final concern regarding the outcome measure is the use of rank-transformed items to handle extreme outliers. This approach preserves information about high-risk individuals and improves distributional properties, but it compresses meaningful variation at the extremes—for example, someone smoking 40 cigarettes versus one may be only one rank apart. Our outcome measure therefore captures relative risk-taking frequency rather than absolute behavioral counts, which limits interpretation of effect magnitudes.

To test the argument that risk-taking frequency is domain-specific rather than general, Bayesian regression analyses were conducted on each of the new indices (see Table S4-S8 in the Supplementary Material). If the variables identified in the study’s main results were not found to be likely predictors when measured against domain-specific risk-taking frequency, this would argue against the use of an aggregated scale. The analyses showed that sensation seeking and impulsivity were important variables for predicting domain-specific risk-taking frequency in three and four of the five cases, respectively (health, social, and safety indices for sensation seeking; health, procrastination, safety, and economic indices for impulsivity). The domain-specific risk preferences for health and social were not important for all indices but most predicted their corresponding indices (health and social). Gender was important in two of the five indices (health and safety). These analyses confirm that the most important predictors, in terms of overall likelihood, are impulsivity and sensation seeking and that domain-specific risk preference are important predictors when measured within its corresponding domain of risk-taking frequency. In none of these analyses was general risk preference found to be a likely predictor for any form of risk-taking frequency. The fact that impulsivity and sensation seeking remained important in most cases when risk-taking frequency was measured across domains supports the validity of an aggregated scale. However, gender, which was presented as important in the main analyses, no longer emerged as likely, highlighting limitations in the use of an aggregated scale.

Another limitation is that we did not include a separate measure of self-control, which is commonly studied alongside impulsivity and sensation seeking in the risk-taking literature^56^. Although impulsivity and self-control are often considered closely related constructs^57^, it is fair to say that they have distinct predictive profiles^58^. Future research should include dedicated measures of both to examine their independent contributions to risk-taking frequency.

Also, our measure of risk-taking frequency relied on self-report, consistent with the ‘frequency measures’ tradition in risk research^14^. Validation studies suggest that self-reported behavioral frequencies show acceptable correspondence with objective criteria in several domains: self-reported smoking shows high concordance with cotinine biomarkers^59,60^, and self-reported alcohol consumption correlates moderately with direct biomarkers^61^. However, correspondence varies across domains and populations, and obtaining objective records across the diverse behaviors assessed here (substance use, traffic incidents, social risks, financial decisions) was not feasible. Future research should further examine the relationship between self-reported risk-taking frequency and objectively verified behavior.

Beyond addressing the limitations noted above, several directions for future research warrant consideration. As noted in the Introduction, risk can be conceptualized from an economic perspective—emphasizing outcome variance—or from a clinical perspective—emphasizing behaviors with potentially harmful consequences- Our operationalization of risk-taking frequency includes items that span both conceptualizations. Some items align more clearly with the economic perspective, such as gambling (which explicitly involves probability distributions) and extreme sports (which involve known injury probabilities). Other items align more with the clinical perspective, capturing behaviors with potential negative consequences where outcome probabilities are less precisely defined, such as procrastination, disclosing confidential information, or expressing dissenting opinions. Many items involve elements of both—for instance, smoking and traffic violations involve both quantifiable outcome variance and potential for harm. While this breadth allowed us to capture a broad ecology of everyday risk-taking behaviors^62^, it also means we cannot determine whether impulsivity, sensation seeking, and domain-specific risk preferences would remain the strongest predictors if risk-taking were operationalized strictly according to one perspective or the other. Future research should examine more closely whether the predictive importance of these variables differs across the economic and clinical conceptualizations of risk.

Relatedly, a distinction can be made between asocial risk-taking—behaviors with consequences primarily for the self—and antisocial risk-taking—behaviors involving potential harm to others (e.g., aggression, criminality). Our scale included items spanning both types, though it was not designed to systematically distinguish between them. Whether asocial and antisocial risk-taking have different predictors, or whether the relative importance of impulsivity, sensation seeking, and risk preferences differs across these categories, is an interesting question for future research.

Finally, future research should seek to leverage objective data to test the claims brought forward here. Our measure of risk-taking frequency relied on self-report, consistent with the ‘frequency measures’ tradition^14^, which captures actual risky activities through retrospective self-assessment.

## Conclusions and implications

The results show that the most important predictors of risk-taking frequency are impulsivity, sensation seeking, health and social risk preferences, and gender. The study highlights the importance of examining multiple variables simultaneously when studying the prediction of risk-taking frequency. The results have potentially important implications for several fields within the social sciences. Much research on risk-taking frequency has focused on risk preferences but should, in the future, broaden its perspective to focus instead on sensation seeking, impulsivity, gender, and the domain-specific risk preferences for health- and social risks. The findings suggest that less emphasis should be placed on individuals stated general attraction to taking risks.

Whether an individual chooses to engage in risk-taking behavior can impact life outcomes and quality of life^63–65^. Therefore, it can be important from a clinical perspective to help individuals either increase or decrease their risk-taking frequency. Clinicians should be aware that individuals who identify as men, those who score high on health and social risk preferences, and individuals with high impulsivity and sensation seeking are generally more likely to accumulate engagement in risk-taking behaviors. A focus on methods to reduce or increase the impact of traits like sensation seeking and impulsivity may, therefore, be useful for individuals struggling with either high or low risk-taking^66^.

## Method

The study design and overarching analysis plan were pre-registered prior to data collection (https://osf.io/54xk3/). The study received approval by meeting all the necessary criteria outlined in the Swedish Act concerning the Ethical Review of Research Involving Humans (2003:460). The research was conducted in accordance with relevant guidelines and regulations, including the Declaration of Helsinki. Informed consents were obtained from all the participants.

### Participants

The selection for the survey used a snowball sampling method in January and February of 2021. The survey was initially distributed to friends and acquaintances, who were asked to share it further with people in their networks. It was also shared in approximately 20 different Facebook groups and emailed to organizations where participants were expected to live in various parts of Sweden. Posters were put up in Stockholm as well. Since the survey was primarily distributed on Swedish platforms, the majority of respondents were expected to reside in Sweden. Participants had to be over 18 years old to take part. A total of 825 individuals responded. Due to insufficient response rates on certain scales (< 80%; see Sect. 2.5, Data Processing), 7.88% (*n* = 65) of the respondents were excluded from the analysis.

After accounting for the exclusions, 66.8% (*n* = 508) identified as women, 33.0% (*n* = 251) as men, 0.1% (*n* = 1) as other, and 0% chose not to answer the question. The average age was 38.4 years (SD = 14.0). The median monthly income was SEK 32,000, and the average income was SEK 45,772 (SD = 75,429), aligning rather closely with general population data [https://www.scb.se/en/finding-statistics/statistics-by-subject-area/labour-market/wages-salaries-and-labour-costs/wage-and-salary-structures-and-employment-in-the-municipalities/pong/tables-and-graphs/average-monthly-salary-by-occupation/]. Regarding education levels, 0.26% (*n* = 2) had no completed education, 3.42% (*n* = 26) had completed elementary school, 25.7% (*n* = 195) had completed high school, 16.1% (*n* = 122) had post-secondary education of less than 3 years, 30.0% (*n* = 228) had post-secondary education of 3 years or more, 21.2% (*n* = 161) had post-secondary education of 5 years or more, and 3.42% (*n* = 26) had doctoral degrees.

As a token of appreciation for participating, respondents could choose to enter a raffle for a gift card worth SEK 500.

## Material

Data collection was conducted using a quantitative survey. The survey was primarily based on previously validated scales. Below, the scales for all variables are presented.

### Demographic variables

Gender was asked based on the gender participants identify with, with the response options being *Woman*, *Man*, and *Other*. Education level was assessed by asking participants about their highest completed education, with seven response options: *No completed education*, *Elementary school*, *High school education*, *Post-secondary education*,* less than 3 years*, *Post-secondary education*,* 3 years or more*, *Post-secondary education*,* 5 years or more*, and *Doctoral degree*. Age and income level were open-ended questions where participants entered their responses numerically. Income was specified as monthly income before taxes.

### Frequency of risk-taking behaviors

A new scale, based on a frequency measure, was developed by the authors to provide a stronger indication of individual engagement in real-life risk-taking behavior and to better highlight differences between individuals compared to using pre-existing scales that often rely on a limited set of response options^21^.

To capture a wide range of real-life risk-taking behavior, 19 questions covering various areas of risk-taking were selected (the questions below are translated from Swedish):

1. How many types of dietary supplements (e.g., vitamins) do you take daily to protect yourself from various types of illnesses? Note: Only supplements not prescribed by a doctor.
2. How much money do you set aside each month as a buffer for “tough times”? Provide the amount in SEK.
3. How many times have you experienced serious consequences due to alcohol? (e.g., being arrested, severe memory loss, hospitalization, relationship problems).
4. How many extreme sports have you tried? (e.g., bungee jumping, skydiving, mountain climbing).
5. How many types of insurance do you have? (e.g., accident insurance, home insurance, income insurance, insurance for specific items like a phone or TV).
6. How many times in the past month have you complained to your boss/teacher?
7. Approximately how many cigarettes have you smoked at most in a single day?
8. How much money do you spend on various forms of gambling each month? (e.g., lottery tickets, betting, online gambling). Provide the amount in SEK.
9. How many times in the past month have you disclosed a secret a friend told you in confidence to someone else?
10. How many times have you been involved in a traffic incident where you were the responsible party? (e.g., hitting an object, crashing a car, bike, or scooter).
11. When the sunny season starts, what SPF (sun protection factor) do you typically use?
12. When the sunny season starts, what SPF should you ideally start with?
13. How many times in the past month have you broken traffic rules? (e.g., speeding, running a red light, parking incorrectly). This applies to both driving and walking.
14. How many times in the past month have you procrastinated work- or school-related tasks until the last minute?
15. How many times in the past year have you avoided seeking medical care at a hospital or clinic despite needing it?
16. How many times in the past month have you expressed a differing opinion in a group?
17. How many types of illegal drugs have you consumed? (e.g., cannabis, ecstasy, cocaine, amphetamines, heroin, or synthetic variants).
18. How many times in the past month have you missed a deadline?
19. How many times in the past month have you started an argument?

Of these, 13 were adapted from a previous pilot-study where most items showed associations with risk preference (see https://osf.io/2cjh3/). That pilot-study also indicated that continuous questions provided stronger regression results compared to binary questions. The items used in the pilot-study included questions about risk-taking frequency in health (e.g., *How many cigarettes have you smoked in a day at most?*), economics (e.g., *How much money do you save each month as a buffer for “tougher times”?*), safety (e.g., *How many times have you been involved in traffic incidents for which you were responsible? Examples: colliding with objects*,* crashing with a car*,* bike*,* or scooter.*), and recreational activities (e.g., *How many extreme sports have you tried? For example*,* bungee jumping*,* skydiving*,* mountain climbing.*).

To further cover additional areas of risk-taking behaviors, six newly developed items were included, focusing on social (e.g., *How many times in the past month have you disclosed something a friend confided to you?*) and career risks (e.g., *How many times in the past month have you missed a deadline?*). These 19 questions were combined into a single measure of risk-taking frequency during the analysis (see Sect. 2.5, Data Processing), which was considered appropriate as previous research has suggested that these or similar behaviors represent typical risk decisions^11,53,62^.

### Risk preference

Both general and domain-specific risk preferences were included. General risk preference was measured using the General Risk Propensity Scale (GRiPSs), which has has demonstrated good reliability and construct validity^67^. Scales measuring domain-specific risk preference, such as DOSPERT^16,68^, have shown good construct validity^68^ and moderate test-retest reliability^16^. However, these scales tend to be “pseudo-naturalistic” as they ask about the likelihood of engaging in a behavior. In this study, the aim was to make a clear distinction between preferences for risks and the actual frequency of behaviors.

To address this, six questions were designed to capture preferences for risks (similar to GRiPS) but tailored to the domains previously studied in DOSPERT (i.e., recreational risks, health risks, career risks, financial risks, safety risks, and social risks). These six questions were phrased as follows:*” I am attracted to risks in the domain of recreational activities/health/career/finance/safety/social situations*” (see the study’s preregistration: https://osf.io/2cjh3/). The scale was translated into Swedish by the authors.

### Anxiety

Anxiety was measured using the short version of the Spielberger State-Trait Anxiety Inventory (STAIT-5^69^, which assesses how anxious the respondent generally feels. The STAIT-5, consisting of five items, has been shown to strongly correlate with the longer version of the scale and demonstrates good internal consistency (α = 0.82)^69^. The scale was translated into Swedish by the authors.

### Sensation seeking

The Brief Sensation Seeking Scale (BSSS^70^; was used to measure sensation seeking. This scale is a shorter version of the widely used Sensation Seeking Scale V (SSS-V^71^;, which has demonstrated strong psychometric properties^32^. The BSSS correlates highly with the SSS-V and has shown good reliability^72^. Other studies have confirmed solid psychometric properties, including adequate internal consistency (α = 0.76), and suggest that the BSSS provides broad coverage of the sensation seeking construct^70^. The scale was translated into Swedish by the authors.

### Impulsivity

Impulsivity was measured using the BIS-15, a shorter version of the BIS-11^73^. The BIS-11 has demonstrated strong test-retest reliability^74^. The BIS-15 demonstrates good internal consistency (α = 0.79–0.81) and correlates strongly with both neurological measures and other scales measuring impulsivity^74,75^. The scale was translated into Swedish by the authors.

### Personality traits: neuroticism and extraversion

For measurement of the personality traits neuroticism and extraversion, two items from these subscales in the NEO-PI-R^76^ were used. The NEO-PI-R has demonstrated good internal consistency (α = 0.85–0.94)^77^, strong temporal stability, and good inter-rater reliability^76^. The scale was translated into Swedish by the authors.

### Data processing

Respondents who left ≥ 20% of the questions unanswered on at least one subscale (*n* = 65, corresponding to 7.88% of all participants) were excluded from the analysis, as internal missingness exceeding 20% is considered problematic^78^. After excluding these 65 participants, the remaining missing data (*n* = 167, corresponding to 0.32% of all responses) were handled using expectation maximization after it was assessed that which was found that the missing data could be considered completely random according to Little’s multivariate test^79^. Expectation maximization was chosen as it better preserves variable variance compared to other methods^80,81^. However, it has been noted that for low levels of internal missingness (< 20%), the choice of imputation method is less critical, as different procedures often yield similar results^78^.

The variable gender, which was measured on a nominal scale, was converted into a dummy variable, where 0 indicated that the participant did not identify as male. To capture participants’ risk-taking frequency regarding sunscreen use, the difference was calculated between the questions “*At the beginning of the sunny season*,* what SPF strength do you typically start with*?” and “*At the beginning of the sunny season*,* what SPF strength should you ideally start with?*” A small difference corresponded to low risk-taking. For the questions regarding saving (“*How much money do you save each month as a buffer for ‘tougher times’? Enter in SEK*”) and gambling (“*How much money do you spend on various types of gambling each month? (e.g.*,* scratch cards*,* horse betting*,* or online gambling)? Enter in SEK*”), the responses were calculated as a percentage of income. A high saving percentage corresponded to low risk-taking, while a high gambling percentage corresponded to high risk-taking. Responses for the number of dietary supplements, the percentage of savings, and the number of insurance policies were reversed so that high scores on all risk-taking questions corre.

Several extreme values (z > 3^82^; were observed for risk-taking frequency and income. Because such values may reflect substantively meaningful cases (e.g., the highest-frequency risk-takers) rather than erroneous observations, we did not exclude them. Instead, to reduce undue leverage of extreme observations and to place variables on a more robust scale, we applied a rank transformation to risk-taking frequency and income—an approach commonly used in regression contexts to improve robustness to outliers and distributional irregularities while preserving ordinal information about participants’ standing^83–85^. For the risk-taking frequency measure, items also differed markedly in units and range (e.g., high-range counts vs. rare events), such that summing raw values would impose an arbitrary weighting scheme in which a few high-variance items could dominate the composite; rank-transforming each item prior to aggregation avoids this problem by putting all indicators on a common scale. After rank-transforming each item on the risk-taking frequency scale, missing data on this scale were imputed using expectation maximization, and each participant’s item ranks were then summed to create a composite index. This composite should be interpreted as capturing relative involvement/standing in risk-related behaviors across indicators rather than absolute behavioral magnitude, which is also consistent with our use of Bayesian model averaging to quantify predictor importance while accounting for model uncertainty rather than to interpret effects in absolute outcome units^86^.

### Statistical analyses

The data met necessary criteria for the use of linear regressions, including little to no autocorrelation, little to no multicollinearity, homoscedasticity, and all variables being multivariate normally distributed^87^.

Autocorrelation was tested using a Durbin-Watson test, which showed a value of 2.09. Multicollinearity was checked as tolerance was > 0.1 and the variance inflation factor (VIF) was < 10. Homoscedasticity was assessed using a residual plot, and multivariate normal distribution was evaluated with a Q-Q plot (see Table S9 and Figure S1−2 in the Supplementary Material).

To address the research questions, Bayesian regression analyses were conducted in JASP, as it provides tools for Bayesian Model Averaging (BMA^88^;. BMA makes it possible to assess both the predictive power and the likelihood of models that include each predictor variable in predicting the outcome variable, while taking all relevant models into account^86,88,89^. In this study, the relevant models included all main effects, resulting in the testing of 65,536 models. Variables were z-transformed to allow for the estimation of standardized regression coefficients. A uniform prior was used.

Only main effects were analyzed, as it is unlikely that including interaction effects would alter the results in a qualitative sense, given that most interactions also involve main effects^79^. BMA also controls for “overfitting,” allowing all variables in the study to be included in the analysis simultaneously without overestimating the variance explained in the true population. This risk is otherwise present in frequentist regression analyses^88^. Including all models simultaneously was advantageous, as the study aimed to examine the extent to which the variables predicted risk-taking frequency across the model space.

Another advantage of Bayesian statistics is that it quantifies each variable’s probability given the data, making it easier to compare variables to one another without relying on effect sizes or p-values^86,90^. For example, consider two models: *M*_*1*_ and *M*_*2*_. In a frequentist framework, both *M*_*1*_ and *M*_*2*_might be reported as significant (*p* =.002), but these p-values do not indicate how probable the models are relative to one another given the data. Instead, they only indicate the likelihood of the data given the null hypothesis. Bayesian statistics, however, allows for the use of Bayes Factors (BF) to quantify what is of interest. For instance, if *M*_*1*_ and *M*_*2*_ have BF_10_ (the probability relative to the null hypothesis) values of 10 and 2, respectively, this indicates that *M*_*1*_ is 5 times more likely than *M*_*2*_ to be the “correct” explanation (10/2 = 5).

To determine which variables were most likely to predict risk-taking frequency, a comparison of the variables’ posterior probabilities was conducted. If the posterior probability of a variable exceeded its prior probability, there is evidence that the variable is an important predictor. A posterior probability close to one indicates that the variable is a critical predictor^91^. Furthermore, the BF specifies how much more likely models that include a variable are compared to those that exclude it.

In the context of BMA, the BF of interest is BF_inclusion_, which states the evidence of how much more likely it is that the data can be explained by models including a term (e.g., impulsivity) as compared to models not including the term.

## Supplementary Information

Below is the link to the electronic supplementary material.

Supplementary Material 1

## Data Availability

The datasets analyzed during the current study are available in the Center for Open Science repository, [https://osf.io/54xk3/].

## References

[1] Aven, T. The risk concept—historical and recent development trends. *Reliab. Eng. Syst. Saf.* **99**, 33–44 (2012).

[2] Schonberg, T., Fox, C. R. & Poldrack, R. A. Mind the gap: bridging economic and naturalistic risk-taking with cognitive neuroscience. *Trends Cogn. Sci.* **15**, 11–19 (2011). 21130018 10.1016/j.tics.2010.10.002 PMC3014440

[3] Knight, F. H. *Risk, Uncertainty and Profit*. Houghton Mifflin (1921).

[4] Carleton, R. N. Fear of the unknown: one fear to rule them all? *J. Anxiety Disord*. **41**, 5–21 (2016). 27067453 10.1016/j.janxdis.2016.03.011

[5] Grupe, D. W. & Nitschke, J. B. Uncertainty and anticipation of threat: an integrative Neurobiological and psychological perspective. *Nat. Rev. Neurosci.* **14**, 488–501 (2013). 23783199 10.1038/nrn3524 PMC4276319

[6] Von Neumann, J. & Morgenstern, O. *Theory of Games and Economic Behavior* 3rd edn (Oxford Univ. Press, 1953).

[7] Kahneman, D. & Tversky, A. Prospect theory: an analysis of decision under risk. *Econometrica* **47**, 263–291 (1979).

[8] Tversky, A. & Kahneman, D. Advances in prospect theory: cumulative representation of uncertainty. *J. Risk Uncertain.* **5**, 297–323 (1992).

[9] Mata, R., Josef, A. K. & Hertwig, R. Propensity for risk taking across the life span and around the Globe. *Psychol. Sci.* **27**, 231–243 (2016). 26744068 10.1177/0956797615617811

[10] Frey, R., Richter, D., Schupp, J., Hertwig, R. & Mata, R. Identifying robust correlates of risk preference: A systematic approach using specification curve analysis. *J Pers. Soc. Psychol. ***120**, 538–557 (2021). 10.1037/pspp0000287 32118465

[11] Brailovskaia, J., Schillack, H., Assion, H., Horn, H. & Margraf, J. Risk-taking propensity and (un)healthy behavior in Germany. *Drug Alcohol Depend.* **192**, 324–328 (2018). 30316034 10.1016/j.drugalcdep.2018.08.027

[12] Fox, C. R. & Tannenbaum, D. The elusive search for stable risk preferences. *Front. Psychol.* **2**, 298 (2011). 22110452 10.3389/fpsyg.2011.00298 PMC3216019

[13] Arslan, R. C. et al. How people know their risk preference. *Sci. Rep.* **10**, 15365 (2020). 32958788 10.1038/s41598-020-72077-5 PMC7505965

[14] Frey, R., Pedroni, A., Mata, R., Rieskamp, J. & Hertwig, R. Risk preference shares the psychometric structure of major psychological traits. *Sci. Adv.* **3**, e1701381 (2017). 28983511 10.1126/sciadv.1701381 PMC5627985

[15] Highhouse, S., Nye, C. D., Zhang, D. C. & Rada, T. B. Structure of the DOSPERT: is there evidence for a general risk factor? *J. Behav. Decis. Mak.* **30**, 400–406 (2017).

[16] Weber, E. U., Blais, A. R. & Betz, N. E. A domain-specific risk-attitude scale: measuring risk perceptions and risk behaviors. *J. Behav. Decis. Mak.* **15**, 263–290 (2002).

[17] Boyer, T. W. The development of risk-taking: A multi-perspective review. *Dev. Rev.* **26**, 291–345 (2006).

[18] Rai, A. A. et al. Relative influences of perceived parental monitoring and perceived peer involvement on adolescent risk behaviors: an analysis of six cross-sectional data sets. *J. Adolesc. Health*. **33**, 108–118 (2003). 12890602 10.1016/s1054-139x(03)00179-4

[19] Casey, B. J., Getz, S. & Galvan, A. The adolescent brain. *Dev. Rev.* **28**, 62–77 (2008). 18688292 10.1016/j.dr.2007.08.003 PMC2500212

[20] Somerville, L. H. & Casey, B. Developmental neurobiology of cognitive control and motivational systems. *Curr. Opin. Neurobiol.* **20**, 236–241 (2010). 20167473 10.1016/j.conb.2010.01.006 PMC3014528

[21] Nicholson, N., Soane, E., Fenton-O’Creevy, M. & Willman, P. Personality and domain-specific risk taking. *J. Risk Res.* **8**, 157–176 (2005).

[22] Byrnes, J. P., Miller, D. C. & Schafer, W. D. Gender differences in risk taking: A meta-analysis. *Psychol. Bull.* **125**, 367–383 (1999).

[23] Wade, J. M. Is it race, sex, gender or all three? Predicting risk for alcohol consumption in emerging adulthood. *J. Child. Fam Stud.* **29**, 3481 (2020).

[24] Peralta, R. L. College alcohol use and the embodiment of hegemonic masculinity among European American men. *Sex. Roles*. **56**, 741–756 (2007).

[25] Atkinson, A. M., Kirton, A. W. & Sumnall, H. R. The gendering of alcohol in consumer magazines: an analysis of male and female targeted publications. *J. Gend. Stud.* **21**, 365–386 (2012).

[26] Bradley, B. J. & Greene, A. C. Do health and education agencies in the united States share responsibility for academic achievement and health? A review of 25 years of evidence about the relationship of adolescents’ academic achievement and health behaviors. *J. Adolesc. Health*. **52**, 523–532 (2013). 23535065 10.1016/j.jadohealth.2013.01.008

[27] De Graaf, H., Vanwesenbeeck, I. & Meijer, S. Educational differences in adolescents’ sexual health: A pervasive phenomenon in a National Dutch sample. *J. Sex. Res.* **52**, 747–757 (2014). 25260077 10.1080/00224499.2014.945111

[28] Donovan, J. E., Jessor, R. & Costa, F. M. Adolescent health behavior and conventionality-unconventionality: an extension of problem-behavior theory. *Health Psychol.* **10**, 52–61 (1991). 2026131

[29] Pfeifer, C. A note on smoking behavior and health risk taking. *Nord J. Health Econ* **1**, 135–151 (2012).

[30] Aguilar, P. et al. The relationships between economic scarcity, concrete mindset and risk behavior: A study of Nicaraguan adolescents. *Int. J. Environ. Res. Public. Health*. **17**, 3845 (2020). 32481716 10.3390/ijerph17113845 PMC7312052

[31] Borhan, M. N., Ibrahim, A. N. H., Aziz, A. & Yazid, M. R. M. The relationship between the demographic, personal, and social factors of Malaysian motorcyclists and risk-taking behavior at signalized intersections. *Accid. Anal. Prev.* **121**, 94–100 (2018). 30237047 10.1016/j.aap.2018.09.004

[32] Mishra, S., Barclay, P. & Sparks, A. The relative state model: integrating need-based and ability-based pathways to risk-taking. *Pers. Soc. Psychol. Rev.* **21**, 176–198 (2017). 27149981 10.1177/1088868316644094

[33] Gambetti, E. & Giusberti, F. The effect of anger and anxiety traits on investment decisions. *J. Econ. Psychol.* **33**, 1059–1069 (2012).

[34] Lorian, C. N. & Grisham, J. R. The safety bias: Risk-avoidance and social anxiety pathology. *Behav. Change*. **27**, 29 (2010).

[35] Auerbach, R. P., Kertz, S. & Gardiner, C. K. Predicting adolescent risky behavior engagement: the role of cognitive vulnerability and anxiety. *Int. J. Cogn. Ther.* **5**, 300–315 (2012).

[36] Cooper, M. L., Agocha, V. B. & Sheldon, M. S. A motivational perspective on risky behaviors: the role of personality and affect regulatory processes. *J. Pers.* **68**, 1059–1088 (2000). 11130732 10.1111/1467-6494.00126

[37] Zuckerman, M. *Sensation Seeking and Risky Behavior* (Am. Psychol. Assoc, 2007).

[38] Fischer, S. & Smith, G. T. Deliberation affects risk-taking beyond sensation seeking. *Pers. Individ Dif*. **36**, 527–537 (2004).

[39] LaSpada, N. et al. Risk-taking, sensation seeking, and personality as related to changes in substance use from adolescence to young adulthood. *J. Adolesc.* **82**, 23–31 (2020). 32512252 10.1016/j.adolescence.2020.04.011 PMC7368834

[40] Dahlen, E. R., Martin, R. C., Ragan, K. & Kuhlman, M. M. Driving anger, sensation seeking, impulsiveness, and boredom proneness in the prediction of unsafe driving. *Accid. Anal. Prev.* **37**, 341–348 (2005). 15667821 10.1016/j.aap.2004.10.006

[41] Cheng, A. S. & Lee, H. C. Risk-taking behavior and response Inhibition of commuter motorcyclists with different levels of impulsivity. *Transp. Res. F Traffic Psychol. Behav.* **15**, 535–543 (2012).

[42] Barratt, E. S., Monahan, J. & Steadman, H. Impulsiveness and aggression. *Violence Ment Disord*. **10**, 61–79 (1994).

[43] Castanier, C., Le Scanff, C. & Woodman, T. Who takes risks in high-risk sports? A typological personality approach. *Res. Q. Exerc. Sport*. **81**, 478–484 (2010). 21268472 10.1080/02701367.2010.10599709

[44] Reynolds, B. W. et al. Executive function, impulsivity, and risky behaviors in young adults. *Neuropsychology* **33**, 212 (2019). 30589284 10.1037/neu0000510

[45] Lejuez, C. W. et al. Evaluation of a behavioral measure of risk-taking: the balloon analogue risk task (BART). *J. Exp. Psychol. Appl.* **8**, 75–84 (2002). 12075692 10.1037//1076-898x.8.2.75

[46] Breivik, G., Sand, T. S. & Sookermany, A. M. Sensation seeking and risk-taking in the Norwegian population. *Pers. Individ Dif*. **119**, 266–272 (2017).

[47] Dohmen, T. et al. Individual risk attitudes: new evidence from a large, representative, experimentally-validated survey. *DIW Discuss. Pap* No. 511 (2005).

[48] Zimerman, L., Shalvi, S. & Bereby-Meyer, Y. Self-reported ethical risk-taking tendencies predict actual dishonesty. *Judgm. Decis. Mak.* **9**, 58–64 (2014).

[49] De Visser, R. O. & Smith, J. A. Alcohol consumption and masculine identity among young men. *Psychol. Health*. **22**, 595–614 (2007).

[50] Dohmen, T. et al. Individual risk attitudes: Measurement, determinants, and behavioral consequences. *J. Eur. Econ. Assoc.* **9**, 522–550 (2011).

[51] Caliendo, M., Fossen, F. M. & Kritikos, A. S. Risk attitudes of nascent entrepreneurs: new evidence from an experimentally validated survey. *Small Bus. Econ.* **32**, 153–167 (2009).

[52] Broman-Fulks, J. J., Urbaniak, A., Bondy, C. L. & Toomey, K. J. Anxiety sensitivity and risk-taking behavior. *Anxiety Stress Coping*. **27**, 619–632 (2014). 24559488 10.1080/10615806.2014.896906

[53] Afzal, S. & Jami, H. Prevalence of academic procrastination and reasons for academic procrastination in university students. *J. Behav. Sci.* **28**, 51–69 (2018).

[54] Conway, J. M. & Huffcutt, A. I. A review and evaluation of exploratory factor analysis practices in organizational research. *Organ. Res. Methods*. **6**, 147–168 (2003).

[55] Bollen, K. A. & Lennox, R. Conventional wisdom on measurement: A structural equation perspective. *Psychol. Bull.* **110**, 305–314 (1991).

[56] Freeman, N. & Muraven, M. Self-control depletion leads to increased risk taking. *Soc. Psychol. Pers. Sci.* **1**, 175–181 (2010).

[57] Duckworth, A. L. & Kern, M. L. A meta-analysis of the convergent validity of self-control measures. *J. Res. Pers.* **45**, 259–268 (2011). 21643479 10.1016/j.jrp.2011.02.004 PMC3105910

[58] Hoyle, R. H. & Gallagher, P. The interplay of personality and self-regulation. In *APA Handb. Pers. Soc. Psychol.* Vol. 4. *Pers. Process. Individ. Differ.* 189–207 (Am. Psychol. Assoc.,) (2015).

[59] Patrick, D. L. et al. The validity of self-reported smoking: A review and meta-analysis. *Am. J. Public. Health*. **84**, 1086–1093 (1994). 8017530 10.2105/ajph.84.7.1086 PMC1614767

[60] Vartiainen, E., Seppälä, T., Lillsunde, P. & Puska, P. Validation of self reported smoking by serum cotinine measurement in a community-based study. *J. Epidemiol. Community Health*. **56**, 167–170 (2002). 11854334 10.1136/jech.56.3.167 PMC1732104

[61] Nielsen, D. G., Andersen, K., Nielsen, A. S., Juhl, C. & Mellentin, A. Consistency between self-reported alcohol consumption and biological markers among patients with alcohol use disorder—a systematic review. *Neurosci. Biobehav Rev.* **124**, 370–385 (2021). 33581224 10.1016/j.neubiorev.2021.02.006

[62] Frey, R. & Fischer, O. Mapping the ecology of risk: 100 risky choices of modern life. *Psychol Sci. ***36**, 846–861 (2025). 10.1177/09567976251384975 PMC13080786 41196690

[63] Buelow, M. *Risky Decision-Making in Psychological Disorders*. Academic Press (2020).

[64] Dickson-Swift, V. A., James, E. L. & Kippen, S. The experience of living with a problem gambler: spouses and partners speak out. *J Gambl. Issues* **13**, 1–22 (2005).

[65] Valois, R. F., Zullig, K. J., Huebner, E. S., Kammermann, S. K. & Drane, J. W. Association between life satisfaction and sexual risk-taking behaviors among adolescents. *J. Child. Fam Stud.* **11**, 427–440 (2002).

[66] Vassileva, J. & Conrod, P. J. Impulsivities and addictions: A multidimensional integrative framework informing assessment and interventions for substance use disorders. *Philos. Trans. R Soc. B Biol. Sci.* **374**, 20180137 (2019). 10.1098/rstb.2018.0137 PMC6335463 30966920

[67] Zhang, D. C., Highhouse, S. & Nye, C. D. Development and validation of the general risk propensity scale (GRiPS). *J. Behav. Decis. Mak.* **32**, 152–167 (2019).

[68] Blais, A. & Weber, E. U. A domain-specific risk-taking (DOSPERT) scale for adult populations. *Judgm. Decis. Mak.* **1**, 33–47 (2006).

[69] Zsido, A. N. et al. Development of the short version of the Spielberger State–Trait anxiety inventory. *Psychiatry Res.* **291**, 113223 (2020). 32563747 10.1016/j.psychres.2020.113223

[70] Hoyle, R. H., Stephenson, M. T., Palmgreen, P., Lorch, E. P. & Donohew, R. L. Reliability and validity of a brief measure of sensation seeking. *Pers. Individ Dif*. **32**, 401–414 (2002).

[71] Zuckerman, M., Eysenck, S. B. & Eysenck, H. J. Sensation seeking in England and america: Cross-cultural, age, and sex comparisons. *J. Consult Clin. Psychol.* **46**, 139 (1978). 627648 10.1037//0022-006x.46.1.139

[72] Litvin, S. W. Sensation seeking and its measurement for tourism research. *J. Travel Res.* **46**, 440–445 (2008).

[73] Spinella, M. Normative data and a short form of the Barratt impulsiveness scale. *Int. J. Neurosci.* **117**, 359–368 (2007). 17365120 10.1080/00207450600588881

[74] Fossati, A., Di Ceglie, A., Acquarini, E. & Barratt, E. S. Psychometric properties of an Italian version of the Barratt impulsiveness Scale–11 (BIS–11) in nonclinical subjects. *J. Clin. Psychol.* **57**, 815–828 (2001). 11344467 10.1002/jclp.1051

[75] Meule, A., Vögele, C. & Kübler, A. Psychometrische Evaluation der deutschen Barratt Impulsiveness Scale–Kurzversion (BIS-15). *Diagnostica* (2011).

[76] Costa, P. T. & McCrae, R. R. Normal personality assessment in clinical practice: the NEO personality inventory. *Psychol. Assess.* **4**, 5–13 (1992).

[77] Källmen, H., Wennberg, P. & Bergman, H. Psychometric properties and norm data of the Swedish version of the NEO-PI-R. *Nord J. Psychiatry*. **65**, 311–314 (2011). 21174492 10.3109/08039488.2010.545433

[78] Peng, C. Y. J., Harwell, M., Liou, S. M. & Ehman, L. H. Advances in missing data methods and implications for educational research. *Real Data Anal.* 3178 (2006).

[79] Little, R. J. A test of missing completely at random for multivariate data with missing values. *J. Am. Stat. Assoc.* **83**, 1198–1202 (1988).

[80] Schlomer, G. L., Bauman, S. & Card, N. A. Best practices for missing data management in counseling psychology. *J. Couns. Psychol.* **57**, 1–10 (2010). 21133556 10.1037/a0018082

[81] Gold, M. S. & Bentler, P. M. Treatments of missing data: A Monte Carlo comparison of RBHDI, iterative stochastic regression imputation, and expectation-maximization. *Struct. Equ Model.* **7**, 319–355 (2000).

[82] Shiffler, R. E. Maximum Z scores and outliers. *Am. Stat.* **42**, 79–80 (1988).

[83] Chen, T., Tang, W., Lu, Y. & Tu, X. Rank regression: an alternative regression approach for data with outliers. *Shanghai Jingshen Yixue*. **26**, 310–315 (2014). 10.11919/j.issn.1002-0829.214148 PMC4248265 25903082

[84] Iman, R. L. & Conover, W. J. The use of the rank transform in regression. *Technometrics* **21**, 499–509 (1979).

[85] Conover, W. J. & Iman, R. L. Rank transformations as a Bridge between parametric and nonparametric statistics. *Am. Stat.* **35**, 124–129 (1981).

[86] Hoeting, J. A., Madigan, D., Raftery, A. E. & Volinsky, C. T. Bayesian model averaging: A tutorial. *Stat. Sci.* **14**, 382–401 (1999).

[87] Goss-Sampson, M. *Statistical Analysis in JASP: A Guide for Students* (2019).

[88] Bergh, D. V. D., Clyde, M. A., Gupta, A. R. K. N., de Jong, T., Gronau, Q. F., Marsman,M., … Wagenmakers, E. J. (2021). A tutorial on Bayesian multi-model linear regression with BAS and JASP. Beh. Res. Meth., 53(6), 2351–2371. 10.3758/s13428-021-01552-2 PMC8613115 33835394

[89] Hinne, M., Gronau, Q. F., van den Bergh, D. & Wagenmakers, E. J. A conceptual introduction to bayesian model averaging. *Adv. Methods Pract. Psychol. Sci.* **3**, 200–215 (2020).

[90] Rouder, J. N., Morey, R. D., Verhagen, J., Swagman, A. R. & Wagenmakers, E. J. Bayesian analysis of factorial designs. *Psychol. Methods*. **22**, 304 (2017). 27280448 10.1037/met0000057

[91] Burnham, K. P. & Anderson, D. R. *Model Selection and Multimodel Inference: A Practical Information-Theoretic Approach* 2nd edn (Springer, 2002).

