# Supplementary material for: General risk preference comes up short when predicting risk-taking frequency
Source: Sci Rep. 2026 Jan 23;16:3049. doi: 10.1038/s41598-026-36713-w (PMC12830746; doi:10.1038/s41598-026-36713-w)
Supplement: Supplementary file 1 — Supplementary Material 1 [file 41598_2026_36713_MOESM1_ESM.docx]

**Predicting Risk-Taking Frequency: General Risk Preference Comes Up Short Against Associated Constructs**

**Authors and Affiliations**

Maja Asp^1^, Marielle Abed^1^, Philip Millroth^1*^

**Supplementary Material**

Supplementary Figure SF1. Residuals vs. Predicted Values3

Supplementary Figure SF2. Q-Q plot of Standardized Residuals4

Supplementary Table S1. Regression Analyses5

Supplementary Table S2. Regression Analyses with Single Predictor6

Supplementary Table S3. Principal Component Analysis7

Supplementary Table S4. Regression Analyses on Index 1.8

Supplementary Table S5. Regression Analyses on Index 2.9

Supplementary Table S6. Regression Analyses on Index 3.10

Supplementary Table S7. Regression Analyses on Index 4.11

Supplementary Table S8. Regression Analyses on Index 5.12

Supplementary Table S9. Multicollinearity checks13

**Supplementary Figure SF1**. Residuals vs. Predicted Values


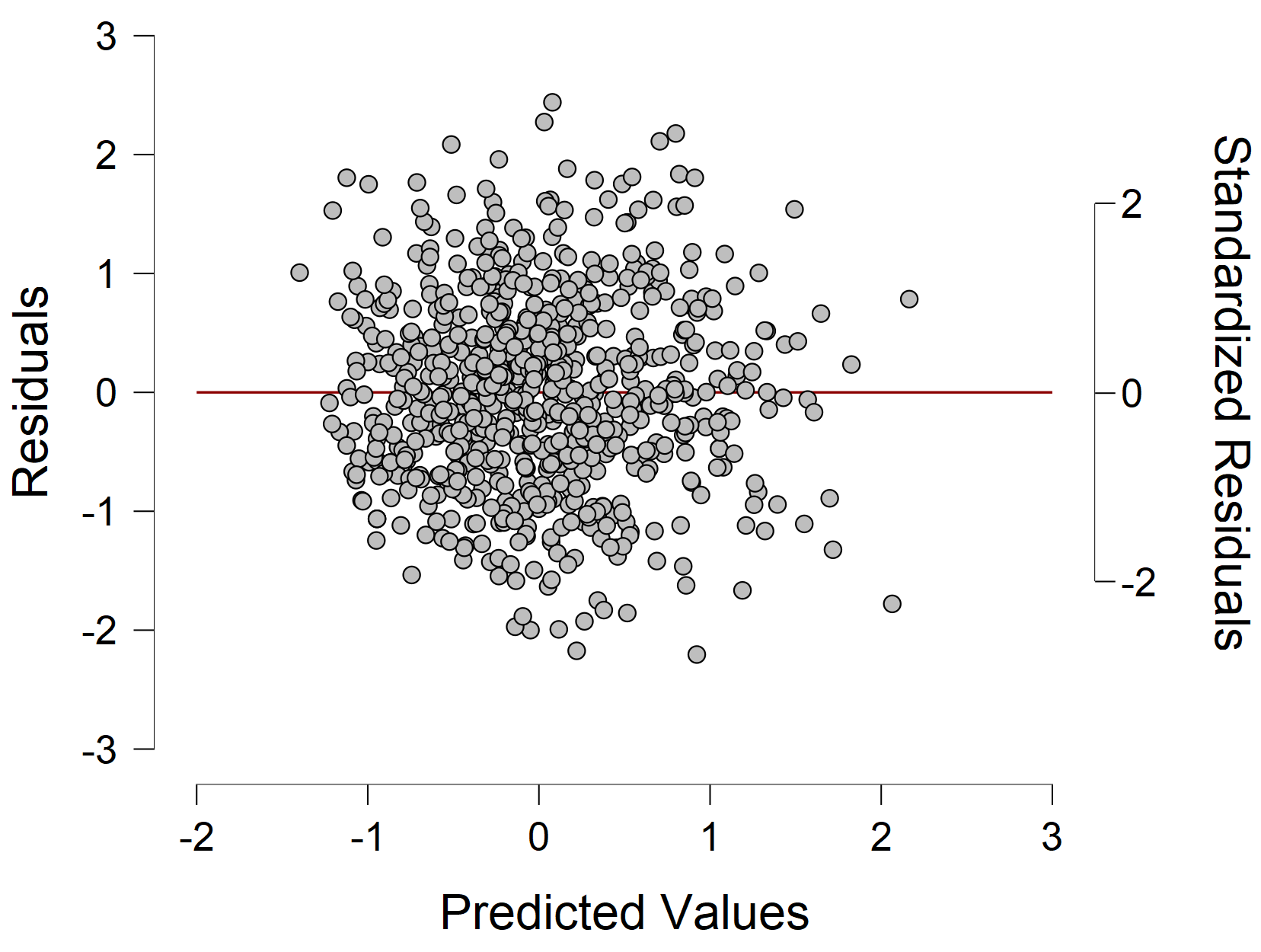


**Supplementary Figure SF2.** Q-Q plot of Standardized Residuals


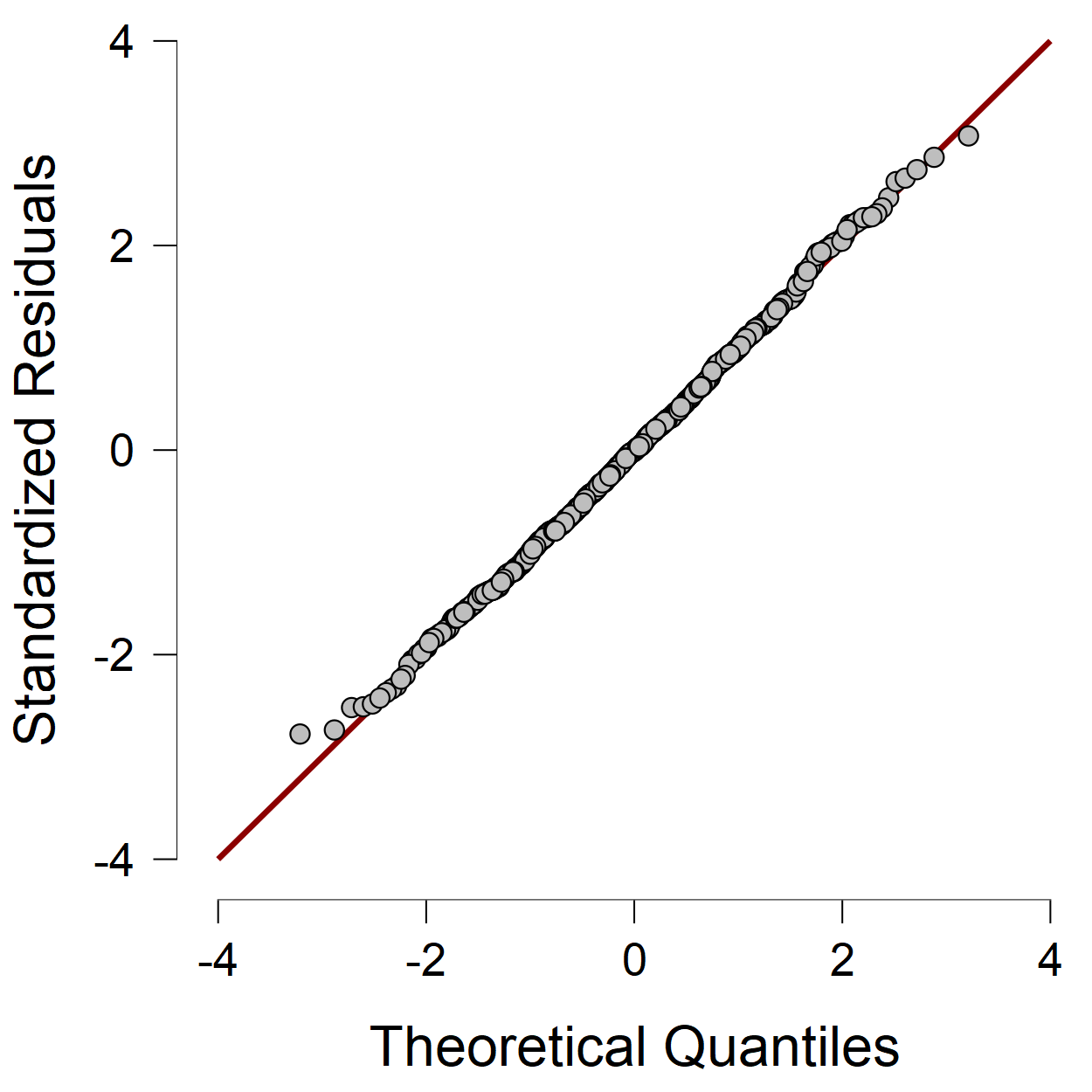


| **Supplementary Table S1.** Detailed results from regression analyses reported in the Results section in the main article. | | | | | | | | | | | | | | | | | | | |
| --- | --- | --- | --- | --- | --- | --- | --- | --- | --- | --- | --- | --- | --- | --- | --- | --- | --- | --- | --- |
|  | | | | | | | | | | | | | | | | 95% Credible Interval | | | |
| Coefficient | | P(incl) | | P(excl) | | P(incl\|data) | | P(excl\|data) | | BF_inclusion_ | | Mean | | SD | | Lower | | Upper | |
| Intercept |  | 1.000 |  | 0.000 |  | 1.000 |  | 0.000 |  | 1.000 |  | 5.186×10^-11^ |  | 0.029 |  | -0.058 |  | 0.056 |  |
| Gender |  | 0.709 |  | 0.291 |  | 0.918 |  | 0.082 |  | 4.563 |  | 0.087 |  | 0.040 |  | 0.000 |  | 0.148 |  |
| Age |  | 0.438 |  | 0.562 |  | 0.321 |  | 0.679 |  | 0.605 |  | -0.018 |  | 0.032 |  | -0.098 |  | 0.000 |  |
| Education |  | 0.318 |  | 0.682 |  | 0.126 |  | 0.874 |  | 0.310 |  | 0.003 |  | 0.013 |  | -0.011 |  | 0.041 |  |
| Income |  | 0.388 |  | 0.612 |  | 0.202 |  | 0.798 |  | 0.401 |  | 0.008 |  | 0.022 |  | 0.000 |  | 0.072 |  |
| General risk preference |  | 0.322 |  | 0.678 |  | 0.125 |  | 0.875 |  | 0.301 |  | -0.003 |  | 0.017 |  | -0.056 |  | 0.007 |  |
| Domain-specific (recreational) |  | 0.321 |  | 0.679 |  | 0.139 |  | 0.861 |  | 0.342 |  | -0.004 |  | 0.019 |  | -0.053 |  | 0.024 |  |
| Domain-specific (health) |  | 0.793 |  | 0.207 |  | 1.000 |  | 2.254×10^-4^ |  | 1159.121 |  | 0.185 |  | 0.035 |  | 0.116 |  | 0.250 |  |
| Domain-specific (career) |  | 0.345 |  | 0.655 |  | 0.161 |  | 0.839 |  | 0.366 |  | 0.006 |  | 0.021 |  | 0.000 |  | 0.069 |  |
| Domain-specific (safety) |  | 0.311 |  | 0.689 |  | 0.114 |  | 0.886 |  | 0.284 |  | 0.002 |  | 0.015 |  | -0.023 |  | 0.042 |  |
| Domain-specific (social) |  | 0.793 |  | 0.207 |  | 0.965 |  | 0.035 |  | 7.112 |  | 0.111 |  | 0.040 |  | 0.045 |  | 0.198 |  |
| Domain-specific (financial) |  | 0.379 |  | 0.621 |  | 0.227 |  | 0.773 |  | 0.483 |  | -0.011 |  | 0.028 |  | -0.087 |  | 0.001 |  |
| Domain-specific (summed) |  | 0.313 |  | 0.687 |  | 0.141 |  | 0.859 |  | 0.360 |  | 0.001 |  | 0.042 |  | -0.125 |  | 0.019 |  |
| Anxiety |  | 0.356 |  | 0.644 |  | 0.183 |  | 0.817 |  | 0.405 |  | 0.008 |  | 0.026 |  | -0.013 |  | 0.078 |  |
| Neuroticism |  | 0.469 |  | 0.531 |  | 0.439 |  | 0.561 |  | 0.887 |  | -0.030 |  | 0.042 |  | -0.119 |  | 8.843×10^-4^ |  |
| Extraversion |  | 0.309 |  | 0.691 |  | 0.116 |  | 0.884 |  | 0.293 |  | 0.002 |  | 0.012 |  | -0.016 |  | 0.036 |  |
| Sensation seeking |  | 0.794 |  | 0.206 |  | 1.000 |  | 1.773×10^-6^ |  | 146696.999 |  | 0.200 |  | 0.036 |  | 0.135 |  | 0.275 |  |
| Impulsivity |  | 0.793 |  | 0.207 |  | 1.000 |  | 1.257×10^-21^ |  | 2.080×10^+20^ |  | 0.338 |  | 0.033 |  | 0.271 |  | 0.398 |  |
|  | | | | | | | | | | | | | | | | | | | |

| **Supplementary Table S2.** Results from analysis when each variable is used as a standalone predictor. | | | | | | | | | | | | | | | | | | | |
| --- | --- | --- | --- | --- | --- | --- | --- | --- | --- | --- | --- | --- | --- | --- | --- | --- | --- | --- | --- |
|  | | | | | | | | | | | | | | | | 95% Credible Interval | | | |
| Coefficient | | P(incl) | | P(excl) | | P(incl\|data) | | P(excl\|data) | | BF_inclusion_ | | Mean | | SD | | Lower | | Upper | |
| Gender |  | 0.500 |  | 0.500 |  | 1.000 |  | 1.117×10^-6^ |  | 895102.060 |  | 0.203 |  | 0.035 |  | 0.142 |  | 0.273 |  |
| Age |  | 0.500 |  | 0.500 |  | 1.000 |  | 2.442×10^-5^ |  | 40954.086 |  | -0.183 |  | 0.035 |  | -0.247 |  | -0.110 |  |
| Education |  | 0.500 |  | 0.500 |  | 0.793 |  | 0.207 |  | 3.826 |  | -0.079 |  | 0.051 |  | -0.154 |  | 0.000 |  |
| Income |  | 0.500 |  | 0.500 |  | 0.217 |  | 0.783 |  | 0.278 |  | -0.012 |  | 0.029 |  | -0.086 |  | 2.502×10^-4^ |  |
| General risk preference |  | 0.500 |  | 0.500 |  | 1.000 |  | 6.311×10^-17^ |  | 1.585×10^+16^ |  | 0.314 |  | 0.034 |  | 0.252 |  | 0.383 |  |
| Domain-specific (recreational) |  | 0.500 |  | 0.500 |  | 1.000 |  | 3.053×10^-6^ |  | 327514.999 |  | 0.197 |  | 0.035 |  | 0.127 |  | 0.260 |  |
| Domain-specific (health) |  | 0.500 |  | 0.500 |  | 1.000 |  | 1.022×10^-22^ |  | 9.781×10^+21^ |  | 0.361 |  | 0.034 |  | 0.298 |  | 0.427 |  |
| Domain-specific (career) |  | 0.500 |  | 0.500 |  | 1.000 |  | 5.434×10^-7^ |  | 1.840×10^+6^ |  | 0.208 |  | 0.035 |  | 0.139 |  | 0.280 |  |
| Domain-specific (safety) |  | 0.500 |  | 0.500 |  | 1.000 |  | 1.339×10^-16^ |  | 7.467×10^+15^ |  | 0.312 |  | 0.034 |  | 0.246 |  | 0.379 |  |
| Domain-specific (social) |  | 0.500 |  | 0.500 |  | 1.000 |  | 1.968×10^-10^ |  | 5.082×10^+9^ |  | 0.251 |  | 0.035 |  | 0.191 |  | 0.323 |  |
| Domain-specific (financial) |  | 0.500 |  | 0.500 |  | 1.000 |  | 8.052×10^-8^ |  | 1.242×10^+7^ |  | 0.219 |  | 0.035 |  | 0.149 |  | 0.285 |  |
| Domain-specific (summed) |  | 0.500 |  | 0.500 |  | 1.000 |  | 6.442×10^-26^ |  | 1.552×10^+25^ |  | 0.383 |  | 0.033 |  | 0.317 |  | 0.451 |  |
| Anxiety |  | 0.500 |  | 0.500 |  | 0.999 |  | 6.213×10^-4^ |  | 1608.564 |  | 0.159 |  | 0.036 |  | 0.094 |  | 0.233 |  |
| Neuroticism |  | 0.500 |  | 0.500 |  | 0.078 |  | 0.922 |  | 0.085 |  | 8.438×10^-4^ |  | 0.010 |  | -0.023 |  | 0.007 |  |
| Extraversion |  | 0.500 |  | 0.500 |  | 0.356 |  | 0.644 |  | 0.554 |  | 0.025 |  | 0.040 |  | 0.000 |  | 0.115 |  |
| Sensation seeking |  | 0.500 |  | 0.500 |  | 1.000 |  | 7.838×10^-31^ |  | 1.276×10^+30^ |  | 0.415 |  | 0.033 |  | 0.355 |  | 0.476 |  |
| Impulsivity |  | 0.500 |  | 0.500 |  | 1.000 |  | 3.901×10^-38^ |  | 2.563×10^+37^ |  | 0.456 |  | 0.032 |  | 0.392 |  | 0.515 |  |
|  | | | | | | | | | | | | | | | | | | | |

**Supplementary Table S3.** Loadings for items that were included in principal component analysis.

| Item | Index 1 | Index 2 | Index 3 | Index 4 | Index 5 | Uniqueness |
| --- | --- | --- | --- | --- | --- | --- |
| 1 |  |  |  |  |  | .921 |
| 2 |  |  |  |  |  | .571 |
| 3 | .690 |  |  |  |  | .493 |
| 4 |  |  |  | .535 |  | .500 |
| 5 |  |  |  |  | -.718 | .460 |
| 6 |  |  | .601 |  |  | .617 |
| 7 | .728 |  |  |  |  | .380 |
| 8 |  |  |  |  | .614 | .605 |
| 9 |  |  |  |  |  | .709 |
| 10 |  |  |  | .633 |  | .597 |
| 11-12 |  |  |  |  |  | .844 |
| 13 |  |  |  | .661 |  | .522 |
| 14 |  | .746 |  |  |  | .401 |
| 15 |  | .500 |  |  |  | .667 |
| 16 |  |  | .752 |  |  | .414 |
| 17 | .836 |  |  |  |  | .333 |
| 18 |  | .724 |  |  |  | .550 |
| 19 |  |  | .717 |  |  | .473 |
| *Note.* Applied rotation method is promax. | | | | | | |

**Supplementary Table S4.** Results from analysis for Index 1.

|  | | | | | | | | | | | | | | | | | | | |
| --- | --- | --- | --- | --- | --- | --- | --- | --- | --- | --- | --- | --- | --- | --- | --- | --- | --- | --- | --- |
|  | | | | | | | | | | | | | | | | 95% Credible Interval | | | |
| Coefficient | | P(incl) | | P(excl) | | P(incl\|data) | | P(excl\|data) | | BF_inclusion_ | | Mean | | SD | | Lower | | Upper | |
| Intercept |  | 1.000 |  | 0.000 |  | 1.000 |  | 0.000 |  | 1.000 |  | 1.128×10^-10^ |  | 0.023 |  | -0.044 |  | 0.043 |  |
| Gender |  | 0.646 |  | 0.354 |  | 0.985 |  | 0.015 |  | 34.854 |  | 0.088 |  | 0.027 |  | 0.038 |  | 0.139 |  |
| Age |  | 0.461 |  | 0.539 |  | 0.267 |  | 0.733 |  | 0.425 |  | -0.010 |  | 0.021 |  | -0.067 |  | 0.002 |  |
| Education |  | 0.447 |  | 0.553 |  | 0.203 |  | 0.797 |  | 0.315 |  | -0.006 |  | 0.016 |  | -0.057 |  | 0.004 |  |
| Income |  | 0.424 |  | 0.576 |  | 0.126 |  | 0.874 |  | 0.195 |  | -7.501×10^-4^ |  | 0.009 |  | -0.028 |  | 0.013 |  |
| General risk preference |  | 0.427 |  | 0.573 |  | 0.134 |  | 0.866 |  | 0.208 |  | -0.001 |  | 0.014 |  | -0.047 |  | 0.010 |  |
| Domain-specific (recreational) |  | 0.504 |  | 0.496 |  | 0.520 |  | 0.480 |  | 1.066 |  | -0.033 |  | 0.041 |  | -0.104 |  | 0.000 |  |
| Domain-specific (health) |  | 0.646 |  | 0.354 |  | 0.999 |  | 0.001 |  | 413.919 |  | 0.270 |  | 0.033 |  | 0.212 |  | 0.335 |  |
| Domain-specific (career) |  | 0.433 |  | 0.567 |  | 0.166 |  | 0.834 |  | 0.260 |  | 0.003 |  | 0.023 |  | -0.025 |  | 0.057 |  |
| Domain-specific (safety) |  | 0.622 |  | 0.378 |  | 0.935 |  | 0.065 |  | 8.734 |  | -0.107 |  | 0.044 |  | -0.172 |  | 0.000 |  |
| Domain-specific (social) |  | 0.431 |  | 0.569 |  | 0.165 |  | 0.835 |  | 0.261 |  | 0.003 |  | 0.023 |  | -0.032 |  | 0.052 |  |
| Domain-specific (financial) |  | 0.444 |  | 0.556 |  | 0.192 |  | 0.808 |  | 0.297 |  | -0.005 |  | 0.020 |  | -0.069 |  | 0.004 |  |
| Domain-specific (summed) |  | 0.462 |  | 0.538 |  | 0.290 |  | 0.710 |  | 0.476 |  | -0.028 |  | 0.081 |  | -0.210 |  | 0.010 |  |
| Anxiety |  | 0.499 |  | 0.501 |  | 0.489 |  | 0.511 |  | 0.962 |  | 0.027 |  | 0.035 |  | 0.000 |  | 0.105 |  |
| Neuroticism |  | 0.449 |  | 0.551 |  | 0.204 |  | 0.796 |  | 0.315 |  | -0.007 |  | 0.021 |  | -0.068 |  | 0.013 |  |
| Extraversion |  | 0.427 |  | 0.573 |  | 0.136 |  | 0.864 |  | 0.210 |  | -0.002 |  | 0.010 |  | -0.037 |  | 0.013 |  |
| Sensation seeking |  | 0.645 |  | 0.355 |  | 1.000 |  | 1.252×10^-12^ |  | 4.398×10^+11^ |  | 0.230 |  | 0.031 |  | 0.166 |  | 0.286 |  |
| Impulsivity |  | 0.644 |  | 0.356 |  | 1.000 |  | 1.546×10^-5^ |  | 35739.808 |  | 0.139 |  | 0.027 |  | 0.086 |  | 0.190 |  |
|  | | | | | | | | | | | | | | | | | | | |

| **Supplementary Table S5.** Results from analysis for Index 2. | | | | | | | | | | | | | | | | | | | |
| --- | --- | --- | --- | --- | --- | --- | --- | --- | --- | --- | --- | --- | --- | --- | --- | --- | --- | --- | --- |
|  | | | | | | | | | | | | | | | | 95% Credible Interval | | | |
| Coefficient | | P(incl) | | P(excl) | | P(incl\|data) | | P(excl\|data) | | BF_inclusion_ | | Mean | | SD | | Lower | | Upper | |
| Intercept |  | 1.000 |  | 0.000 |  | 1.000 |  | 0.000 |  | 1.000 |  | -2.372×10^-11^ |  | 0.023 |  | -0.043 |  | 0.051 |  |
| Gender |  | 0.414 |  | 0.586 |  | 0.161 |  | 0.839 |  | 0.272 |  | -0.002 |  | 0.011 |  | -0.032 |  | 0.020 |  |
| Age |  | 0.693 |  | 0.307 |  | 0.996 |  | 0.004 |  | 115.640 |  | -0.102 |  | 0.027 |  | -0.152 |  | -0.044 |  |
| Education |  | 0.642 |  | 0.358 |  | 0.922 |  | 0.078 |  | 6.615 |  | 0.070 |  | 0.032 |  | 0.000 |  | 0.121 |  |
| Income |  | 0.454 |  | 0.546 |  | 0.294 |  | 0.706 |  | 0.503 |  | 0.011 |  | 0.023 |  | -0.007 |  | 0.065 |  |
| General risk preference |  | 0.438 |  | 0.562 |  | 0.232 |  | 0.768 |  | 0.388 |  | 0.008 |  | 0.024 |  | -0.015 |  | 0.079 |  |
| Domain-specific (recreational) |  | 0.481 |  | 0.519 |  | 0.400 |  | 0.600 |  | 0.718 |  | -0.022 |  | 0.035 |  | -0.108 |  | 0.002 |  |
| Domain-specific (health) |  | 0.466 |  | 0.534 |  | 0.335 |  | 0.665 |  | 0.579 |  | 0.014 |  | 0.026 |  | 0.000 |  | 0.082 |  |
| Domain-specific (career) |  | 0.466 |  | 0.534 |  | 0.336 |  | 0.664 |  | 0.580 |  | 0.014 |  | 0.027 |  | -0.002 |  | 0.090 |  |
| Domain-specific (safety) |  | 0.419 |  | 0.581 |  | 0.174 |  | 0.826 |  | 0.291 |  | 1.508×10^-4^ |  | 0.015 |  | -0.030 |  | 0.042 |  |
| Domain-specific (social) |  | 0.436 |  | 0.564 |  | 0.230 |  | 0.770 |  | 0.387 |  | 0.006 |  | 0.020 |  | -0.018 |  | 0.078 |  |
| Domain-specific (financial) |  | 0.432 |  | 0.568 |  | 0.209 |  | 0.791 |  | 0.348 |  | 0.005 |  | 0.018 |  | -0.013 |  | 0.060 |  |
| Domain-specific (summed) |  | 0.513 |  | 0.487 |  | 0.555 |  | 0.445 |  | 1.186 |  | 0.050 |  | 0.058 |  | 0.000 |  | 0.161 |  |
| Anxiety |  | 0.527 |  | 0.473 |  | 0.629 |  | 0.371 |  | 1.523 |  | 0.041 |  | 0.041 |  | 0.000 |  | 0.124 |  |
| Neuroticism |  | 0.471 |  | 0.529 |  | 0.353 |  | 0.647 |  | 0.613 |  | -0.017 |  | 0.031 |  | -0.095 |  | 0.006 |  |
| Extraversion |  | 0.443 |  | 0.557 |  | 0.244 |  | 0.756 |  | 0.407 |  | -0.007 |  | 0.017 |  | -0.051 |  | 0.009 |  |
| Sensation seeking |  | 0.478 |  | 0.522 |  | 0.400 |  | 0.600 |  | 0.728 |  | -0.019 |  | 0.030 |  | -0.088 |  | 0.005 |  |
| Impulsivity |  | 0.693 |  | 0.307 |  | 1.000 |  | 4.048×10^-15^ |  | 1.093×10^+14^ |  | 0.231 |  | 0.027 |  | 0.180 |  | 0.291 |  |
|  | | | | | | | | | | | | | | | | | | | |

| **Supplementary Table S6.** Results from analysis for Index 3. | | | | | | | | | | | | | | | | | | | |
| --- | --- | --- | --- | --- | --- | --- | --- | --- | --- | --- | --- | --- | --- | --- | --- | --- | --- | --- | --- |
|  | | | | | | | | | | | | | | | | 95% Credible Interval | | | |
| Coefficient | | P(incl) | | P(excl) | | P(incl\|data) | | P(excl\|data) | | BF_inclusion_ | | Mean | | SD | | Lower | | Upper | |
| Intercept |  | 1.000 |  | 0.000 |  | 1.000 |  | 0.000 |  | 1.000 |  | -2.259×10^-10^ |  | 0.024 |  | -0.054 |  | 0.044 |  |
| Gender |  | 0.453 |  | 0.547 |  | 0.287 |  | 0.713 |  | 0.485 |  | 0.010 |  | 0.021 |  | -4.737×10^-4^ |  | 0.065 |  |
| Age |  | 0.414 |  | 0.586 |  | 0.161 |  | 0.839 |  | 0.271 |  | 1.023×10^-4^ |  | 0.011 |  | -0.022 |  | 0.030 |  |
| Education |  | 0.419 |  | 0.581 |  | 0.166 |  | 0.834 |  | 0.277 |  | 3.469×10^-4^ |  | 0.012 |  | -0.025 |  | 0.034 |  |
| Income |  | 0.519 |  | 0.481 |  | 0.588 |  | 0.412 |  | 1.326 |  | 0.031 |  | 0.033 |  | 0.000 |  | 0.092 |  |
| General risk preference |  | 0.424 |  | 0.576 |  | 0.186 |  | 0.814 |  | 0.310 |  | -0.004 |  | 0.020 |  | -0.074 |  | 0.019 |  |
| Domain-specific (recreational) |  | 0.538 |  | 0.462 |  | 0.677 |  | 0.323 |  | 1.801 |  | -0.054 |  | 0.048 |  | -0.137 |  | 0.000 |  |
| Domain-specific (health) |  | 0.454 |  | 0.546 |  | 0.279 |  | 0.721 |  | 0.467 |  | -0.009 |  | 0.024 |  | -0.071 |  | 0.003 |  |
| Domain-specific (career) |  | 0.424 |  | 0.576 |  | 0.187 |  | 0.813 |  | 0.312 |  | 0.002 |  | 0.018 |  | -0.042 |  | 0.041 |  |
| Domain-specific (safety) |  | 0.432 |  | 0.568 |  | 0.215 |  | 0.785 |  | 0.359 |  | -0.005 |  | 0.021 |  | -0.076 |  | 0.004 |  |
| Domain-specific (social) |  | 0.696 |  | 0.304 |  | 0.999 |  | 5.727×10^-4^ |  | 761.733 |  | 0.231 |  | 0.041 |  | 0.150 |  | 0.307 |  |
| Domain-specific (financial) |  | 0.429 |  | 0.571 |  | 0.200 |  | 0.800 |  | 0.333 |  | 0.004 |  | 0.020 |  | -0.030 |  | 0.063 |  |
| Domain-specific (summed) |  | 0.494 |  | 0.506 |  | 0.468 |  | 0.532 |  | 0.903 |  | -0.051 |  | 0.075 |  | -0.229 |  | 0.000 |  |
| Anxiety |  | 0.420 |  | 0.580 |  | 0.169 |  | 0.831 |  | 0.282 |  | -0.001 |  | 0.013 |  | -0.037 |  | 0.029 |  |
| Neuroticism |  | 0.443 |  | 0.557 |  | 0.244 |  | 0.756 |  | 0.404 |  | -0.007 |  | 0.019 |  | -0.062 |  | 0.000 |  |
| Extraversion |  | 0.532 |  | 0.468 |  | 0.656 |  | 0.344 |  | 1.679 |  | 0.037 |  | 0.034 |  | 0.000 |  | 0.092 |  |
| Sensation seeking |  | 0.696 |  | 0.304 |  | 0.995 |  | 0.005 |  | 80.268 |  | 0.118 |  | 0.033 |  | 0.054 |  | 0.184 |  |
| Impulsivity |  | 0.550 |  | 0.450 |  | 0.734 |  | 0.266 |  | 2.263 |  | 0.048 |  | 0.038 |  | -0.003 |  | 0.107 |  |
|  | | | | | | | | | | | | | | | | | | | |

| **Supplementary Table S7.** Results from analysis for Index 4. | | | | | | | | | | | | | | | | | | | |
| --- | --- | --- | --- | --- | --- | --- | --- | --- | --- | --- | --- | --- | --- | --- | --- | --- | --- | --- | --- |
|  | | | | | | | | | | | | | | | | 95% Credible Interval | | | |
| Coefficient | | P(incl) | | P(excl) | | P(incl\|data) | | P(excl\|data) | | BF_inclusion_ | | Mean | | SD | | Lower | | Upper | |
| Intercept |  | 1.000 |  | 0.000 |  | 1.000 |  | 0.000 |  | 1.000 |  | -2.235×10^-11^ |  | 0.022 |  | -0.049 |  | 0.041 |  |
| Gender |  | 0.481 |  | 0.519 |  | 0.403 |  | 0.597 |  | 0.728 |  | 0.015 |  | 0.024 |  | -0.007 |  | 0.071 |  |
| Age |  | 0.479 |  | 0.521 |  | 0.385 |  | 0.615 |  | 0.683 |  | 0.014 |  | 0.024 |  | -0.001 |  | 0.073 |  |
| Education |  | 0.480 |  | 0.520 |  | 0.404 |  | 0.596 |  | 0.732 |  | -0.016 |  | 0.025 |  | -0.082 |  | 0.001 |  |
| Income |  | 0.687 |  | 0.313 |  | 1.000 |  | 9.090×10^-6^ |  | 50131.949 |  | -0.136 |  | 0.026 |  | -0.191 |  | -0.084 |  |
| General risk preference |  | 0.496 |  | 0.504 |  | 0.478 |  | 0.522 |  | 0.929 |  | -0.026 |  | 0.035 |  | -0.107 |  | 1.610×10^-4^ |  |
| Domain-specific (recreational) |  | 0.451 |  | 0.549 |  | 0.270 |  | 0.730 |  | 0.450 |  | -0.008 |  | 0.023 |  | -0.077 |  | 0.012 |  |
| Domain-specific (health) |  | 0.613 |  | 0.387 |  | 0.897 |  | 0.103 |  | 5.512 |  | 0.067 |  | 0.035 |  | -0.001 |  | 0.122 |  |
| Domain-specific (career) |  | 0.432 |  | 0.568 |  | 0.200 |  | 0.800 |  | 0.329 |  | -0.001 |  | 0.016 |  | -0.044 |  | 0.034 |  |
| Domain-specific (safety) |  | 0.436 |  | 0.564 |  | 0.217 |  | 0.783 |  | 0.359 |  | -0.004 |  | 0.018 |  | -0.060 |  | 0.013 |  |
| Domain-specific (social) |  | 0.441 |  | 0.559 |  | 0.237 |  | 0.763 |  | 0.392 |  | -0.005 |  | 0.020 |  | -0.066 |  | 0.019 |  |
| Domain-specific (financial) |  | 0.605 |  | 0.395 |  | 0.885 |  | 0.115 |  | 5.041 |  | 0.070 |  | 0.039 |  | 0.000 |  | 0.133 |  |
| Domain-specific (summed) |  | 0.467 |  | 0.533 |  | 0.339 |  | 0.661 |  | 0.583 |  | -0.014 |  | 0.056 |  | -0.181 |  | 0.067 |  |
| Anxiety |  | 0.623 |  | 0.377 |  | 0.906 |  | 0.094 |  | 5.850 |  | -0.069 |  | 0.034 |  | -0.123 |  | 4.511×10^-4^ |  |
| Neuroticism |  | 0.435 |  | 0.565 |  | 0.219 |  | 0.781 |  | 0.365 |  | -0.004 |  | 0.017 |  | -0.062 |  | 0.024 |  |
| Extraversion |  | 0.523 |  | 0.477 |  | 0.619 |  | 0.381 |  | 1.480 |  | -0.029 |  | 0.029 |  | -0.087 |  | 0.000 |  |
| Sensation seeking |  | 0.427 |  | 0.573 |  | 0.186 |  | 0.814 |  | 0.308 |  | 1.879×10^-4^ |  | 0.013 |  | -0.032 |  | 0.031 |  |
| Impulsivity |  | 0.676 |  | 0.324 |  | 0.968 |  | 0.032 |  | 14.357 |  | 0.077 |  | 0.029 |  | 0.000 |  | 0.122 |  |
|  | | | | | | | | | | | | | | | | | | | |

| **Supplementary Table S8.** Results from analysis for Index 5. | | | | | | | | | | | | | | | | | | | |
| --- | --- | --- | --- | --- | --- | --- | --- | --- | --- | --- | --- | --- | --- | --- | --- | --- | --- | --- | --- |
|  | | | | | | | | | | | | | | | | 95% Credible Interval | | | |
| Coefficient | | P(incl) | | P(excl) | | P(incl\|data) | | P(excl\|data) | | BF_inclusion_ | | Mean | | SD | | Lower | | Upper | |
| Intercept |  | 1.000 |  | 0.000 |  | 1.000 |  | 0.000 |  | 1.000 |  | 1.128×10^-10^ |  | 0.023 |  | -0.044 |  | 0.043 |  |
| Gender |  | 0.646 |  | 0.354 |  | 0.985 |  | 0.015 |  | 34.854 |  | 0.088 |  | 0.027 |  | 0.038 |  | 0.139 |  |
| Age |  | 0.461 |  | 0.539 |  | 0.267 |  | 0.733 |  | 0.425 |  | -0.010 |  | 0.021 |  | -0.067 |  | 0.002 |  |
| Education |  | 0.447 |  | 0.553 |  | 0.203 |  | 0.797 |  | 0.315 |  | -0.006 |  | 0.016 |  | -0.057 |  | 0.004 |  |
| Income |  | 0.424 |  | 0.576 |  | 0.126 |  | 0.874 |  | 0.195 |  | -7.501×10^-4^ |  | 0.009 |  | -0.028 |  | 0.013 |  |
| General risk preference |  | 0.427 |  | 0.573 |  | 0.134 |  | 0.866 |  | 0.208 |  | -0.001 |  | 0.014 |  | -0.047 |  | 0.010 |  |
| Domain-specific (recreational) |  | 0.504 |  | 0.496 |  | 0.520 |  | 0.480 |  | 1.066 |  | -0.033 |  | 0.041 |  | -0.104 |  | 0.000 |  |
| Domain-specific (health) |  | 0.646 |  | 0.354 |  | 0.999 |  | 0.001 |  | 413.919 |  | 0.270 |  | 0.033 |  | 0.212 |  | 0.335 |  |
| Domain-specific (career) |  | 0.433 |  | 0.567 |  | 0.166 |  | 0.834 |  | 0.260 |  | 0.003 |  | 0.023 |  | -0.025 |  | 0.057 |  |
| Domain-specific (safety) |  | 0.622 |  | 0.378 |  | 0.935 |  | 0.065 |  | 8.734 |  | -0.107 |  | 0.044 |  | -0.172 |  | 0.000 |  |
| Domain-specific (social) |  | 0.431 |  | 0.569 |  | 0.165 |  | 0.835 |  | 0.261 |  | 0.003 |  | 0.023 |  | -0.032 |  | 0.052 |  |
| Domain-specific (financial) |  | 0.444 |  | 0.556 |  | 0.192 |  | 0.808 |  | 0.297 |  | -0.005 |  | 0.020 |  | -0.069 |  | 0.004 |  |
| Domain-specific (summed) |  | 0.462 |  | 0.538 |  | 0.290 |  | 0.710 |  | 0.476 |  | -0.028 |  | 0.081 |  | -0.210 |  | 0.010 |  |
| Anxiety |  | 0.499 |  | 0.501 |  | 0.489 |  | 0.511 |  | 0.962 |  | 0.027 |  | 0.035 |  | 0.000 |  | 0.105 |  |
| Neuroticism |  | 0.449 |  | 0.551 |  | 0.204 |  | 0.796 |  | 0.315 |  | -0.007 |  | 0.021 |  | -0.068 |  | 0.013 |  |
| Extraversion |  | 0.427 |  | 0.573 |  | 0.136 |  | 0.864 |  | 0.210 |  | -0.002 |  | 0.010 |  | -0.037 |  | 0.013 |  |
| Sensation seeking |  | 0.645 |  | 0.355 |  | 1.000 |  | 1.252×10^-12^ |  | 4.398×10^+11^ |  | 0.230 |  | 0.031 |  | 0.166 |  | 0.286 |  |
| Impulsivity |  | 0.644 |  | 0.356 |  | 1.000 |  | 1.546×10^-5^ |  | 35739.808 |  | 0.139 |  | 0.027 |  | 0.086 |  | 0.190 |  |
|  | | | | | | | | | | | | | | | | | | | |

**Supplementary Table S9.** *Tolerance and Variance Inflation Factor for all Predictors.*

|  | Collinearity statistic | |
| --- | --- | --- |
| Prediktor | Tolerance | VIF |
| Gender | .777 | 1.29 |
| Age | .717 | 1.39 |
| Education | .721 | 1.39 |
| Income | .693 | 1.44 |
| General risk preference | .360 | 2.78 |
| Domain-specific (recreational) | .532 | 1.88 |
| Domain-specific (health) | .711 | 1.41 |
| Domain-specific (career) | .647 | 1.55 |
| Domain-specific (safety) | .564 | 1.77 |
| Domain-specific (social) | .655 | 1.53 |
| Domain-specific (financial) | .608 | 1.65 |
| Anxiety | .493 | 2.03 |
| Neuroticism | .528 | 1.89 |
| Extraversion | .818 | 1.22 |
| Sensation seeking | .561 | 1.78 |
| Impulsivity | .697 | 1.44 |
